# A Systematic Review on Evaluating Responsiveness of Parent- or Caregiver-Reported Child Maltreatment Measures for Interventions

**DOI:** 10.1177/15248380221093690

**Published:** 2022-05-22

**Authors:** Sangwon Yoon, Renée Speyer, Reinie Cordier, Pirjo Aunio, Airi Hakkarainen

**Affiliations:** 1 Department of Special Needs Education, Faculty of Education, 205777 University of Oslo, Oslo, Norway; 2 Curtin School of Allied Health, Faculty of Health Sciences, 168274 Curtin University, Perth, WA, Australia; 3 Department of Otorhinolaryngology and Head and Neck Surgery, 4501 Leiden University Medical Centre, Leiden, Netherlands; 4 Department of Social Work, Education and Community Wellbeing, Faculty of Health and Life Sciences, 573762 Northumbria University, Newcastle upon Tyne, UK; 5 Department of Education, Faculty of Educational Sciences, 121561 University of Helsinki, Helsinki, Finland; 6 Open University, 3835 University of Helsinki, Helsinki, Finland

**Keywords:** assessment, child abuse, COnsensus-based Standards for the selection of health Measurement INstruments, measure, parent report, measurement properties, responsiveness

## Abstract

**Aims:** Child maltreatment (CM) is a global public health and social problem, resulting in serious long-term health and socioeconomic consequences. As parents are the most common perpetrators of CM, parenting interventions are appropriate strategies to prevent CM. However, research on parenting interventions on CM has been hampered by lack of consensus on what measures are most responsive to detect a reduction in parental maltreating behaviours after parenting intervention. This systematic review aimed to evaluate the responsiveness of all current parent- or caregiver-reported CM measures. **Methods:** A systematic search was conducted in CINAHL, Embase, ERIC, PsycINFO, PubMed and Sociological Abstracts. The quality of studies and responsiveness of the measures were evaluated using the COnsensus-based Standards for the selection of health Measurement INstruments (COSMIN) guidelines for systematic reviews of patient-reported outcome measures. Only measures developed and published in English were included. Studies reporting data on responsiveness of the included measures were selected. **Results:** Sixty-nine articles reported on responsiveness of 15 identified measures. The study quality was overall adequate. The responsiveness of the measures was overall insufficient or not reported; high-quality evidence on responsiveness was limited. **Conclusions:** Only the Physical Abuse subscale of the ISPCAN Child Abuse Screening Tool for use in Trials (ICAST-Trial) can be recommended as most responsive for use in parenting interventions, with high-quality evidence supporting sufficient responsiveness. All other overall scales or subscales of the 15 included measures were identified as promising based on current data on responsiveness. Additional psychometric evidence is required before they can be recommended.

## Introduction

Child maltreatment (CM) refers to the abuse and neglect experienced by a child under the age of 18 years, resulting in actual or potential harm to the child (World Health Organization [WHO], 2016). This conceptual definition can be categorised into four subtypes of CM (Slep et al., 2015; WHO, 2006): (1) physical abuse (non-accidental acts of physical force causing actual or potential physical harm); (2) emotional abuse (non-accidental verbal or symbolic acts causing significant psychological harm); (3) sexual abuse (sexual acts using a child for sexual gratification) and (4) neglect (failure in providing a child with needed age-appropriate care in health, education, emotional development, nutrition, shelter and safe living conditions).

Child maltreatment is a pervasive public health problem and societal burden. Worldwide, more than 1 billion children (aged 2–17 years) are annually exposed to at least one type of CM (Hillis et al., 2016); a minimum of 64% of children in Asia, 56% in North America, 50% in Africa, 34% in Latin America and 12% in Europe were exposed to some form of violence in the past year (Hillis et al., 2016). Early exposure to multiple types and repeated episodes of CM can cause childhood adverse outcomes such as physical injuries, mental health problems and death (Coley et al., 2014; Gilbert et al., 2009; Louwers et al., 2011; MacKenzie et al., 2015). Childhood physical and mental health problems due to exposure to CM can also persist into adulthood and cause adverse outcomes such as chronic diseases, depression, substance use and suicidal behaviour (Currie & Widom, 2010; Hughes et al., 2017). Furthermore, CM is associated with high economic burden. For example, the lifetime estimated financial cost for each victim of CM is approximately USD 210,012 which is higher than other costly health conditions such as stroke (USD 159,846) or type 2 diabetes (USD 181,000; Fang et al., 2012). Given the great health and societal impact of CM, the importance of preventing CM cannot be overstated.

As parents comprise the majority of CM perpetrators (e.g. every year more than 80% of CM perpetrators in the US are parents, Devries et al., 2018; Petersen et al., 2014; Sedlak et al., 2010), parenting interventions are one of the main strategies used to prevent CM (Hinds & Giardino, 2017; WHO, 2016). Parenting interventions provide parents with a comprehensive support service to reduce risk factors (e.g. parental mental health disorders related to their childhood maltreatment experience) and enhance protective factors (e.g. positive parenting behaviour, attitude and relationship between parents and their children) for CM (Austin et al., 2020; Chen & Chan, 2016; Temcheff et al., 2018). Parenting interventions usually include individual or group-based support services in terms of the service delivery method (Chen & Chan, 2016). For example, home visits are a typical individual parenting intervention in which professionals offer one-to-one services in observing and teaching strategies to discipline children, while parent education, which aims to improve parents’ knowledge and attitude regarding parenting strategies or child behaviours, is usually provided in clinics or service centres via group training. Regardless of whether the service delivery method is individual or group interventions, parenting interventions are effective in reducing CM according to Chen and Chan (2016). They conducted a meta-analysis which compared effectiveness in accordance with the characteristics of study (e.g. sample size) and intervention (e.g. intervention dosage).

Research on parenting interventions to reduce CM is hampered by the lack of consensus on which CM measures is most responsive to detecting treatment effects following interventions for reducing CM by parents (Fluke et al., 2020). To draw valid conclusions about the effectiveness of treatments, efficacy studies on CM interventions should use CM measures appropriate and sensitive enough to detect changes in parenting behaviour before and after parenting interventions (Mokkink et al., 2021). However, many CM efficacy studies used indirect measures (e.g. measures evaluating parental depression and parental stress) that do not capture actual reductions in CM (Mikton & Butchart, 2009) and parent survey measures (e.g. measures estimating prevalence of CM) that may be less sensitive to measure actual reductions in parental maltreating behaviours in intervention studies (Cluver et al., 2016). Furthermore, some studies used CM observational measures (i.e. outsiders’ observation parenting behaviours) that cannot capture extreme cases of parental maltreating behaviours, such as using harsh physical discipline (Presser & Stinson, 1998) and leaving a child at home without supervision (Singer et al., 1995). Furthermore, they are considerably more complex, costly and time-consuming to administer compared with parent report measures (Morsbach & Prinz, 2006). However, the accuracy of parents reporting on their own perpetration of CM is also controversial as parents tend to respond in socially desirable ways (i.e. social desirability bias; Milner & Crouch, 1997) and struggle remembering past events (i.e. recall bias, Greenhoot, 2013). Therefore, identifying high-quality parent- or caregiver-reported measures that are sensitive enough to measure change over time in response to a parenting intervention, is essential to detect intervention effects accurately.

The quality of a measure is largely determined by its psychometric properties (Karanicolas et al., 2009) and consists of the following three overarching constructs: validity (the extent to which a measure assesses the construct it is intended to assess), reliability (the extent to which scores for patients who have not changed are the same for repeated assessments) and responsiveness (the ability to detect change over time in the construct measured; Prinsen et al., 2018). The best way for selecting the most valid, reliable and responsive measures is to systematically review the psychometric properties of existing measures (Scholtes et al., 2011). Recently, the COnsensus-based Standards for the selection of health Measurement INstruments (COSMIN) group has updated comprehensive guidelines for conducting systematic reviews on psychometric properties of health measures (Prinsen et al., 2018; Terwee et al., 2018). The COSMIN guidelines provide the following useful tools: a taxonomy on terms and definitions of each psychometric property (Mokkink et al., 2010b); a checklist for assessing the methodological quality of psychometric studies (Mokkink, de Vet, et al., 2018); quality criteria for evaluating single study results on a psychometric property (Prinsen et al., 2018; Terwee et al., 2018) and a rating system summarising all study results on each psychometric property and grading quality of all evidence used for assessing both the methodological and the psychometric quality (Prinsen et al., 2018; Terwee et al., 2018).

For evaluating responsiveness, the COSMIN guidelines suggest testing the following two approaches: criterion and construct (Mokkink, Prinsen, et al., 2018; Prinsen et al., 2018). The criterion approach assesses the relationship of change scores between the measures and a gold standard (i.e. a single error-free reference measure; Naaktgeboren et al., 2013) for detecting the effect of intervention for preventing CM (i.e. comparison to a gold standard; Mokkink, Prinsen, et al., 2018). If there is no gold standard assessment available, as is the case of measuring the construct CM (Bailhache et al., 2013), the COSMIN guidelines (Mokkink, Prinsen, et al., 2018) recommend using the construct approach instead. The construct approach assesses the following three aspects: (1) the relationship between the change scores on the reviewed measures and other measures used to assess the same construct (i.e. comparison with other outcome measures); (2) the mean difference in change scores for measures between different subgroups (i.e. comparison between subgroups) and (3) the mean difference in change scores for measures before and after intervention (i.e. comparison before and after intervention).

Only one systematic review to date has evaluated responsiveness of CM measures (Saini et al., 2019), which identified child or clinician report CM measures and evaluated the measures’ responsiveness. However, the authors did not include parent- or caregiver-reported measures. Furthermore, the authors did not use the recently revised COSMIN guidelines (Prinsen et al., 2018; Terwee et al., 2018), but old versions of the COSMIN checklist (Mokkink et al., 2010a) and quality criteria (Terwee et al., 2007) to assess the methodological quality of included studies and the responsiveness of measures. These older versions of the checklist and quality criteria have neither a standardised method for summarising evidence on each psychometric property including responsiveness, nor for grading quality of evidence when deciding whether to recommend a measure for research and clinical use (Prinsen et al., 2018; Terwee et al., 2018). To overcome these limitations of older versions, the COSMIN guidelines have been thoroughly revised in recent years (Prinsen et al., 2018; Terwee et al., 2018).

Yoon et al. (2021a, 2021b published two psychometric reviews on parent- or caregiver-reported measures on CM using the latest versions of the COSMIN guidelines (Prinsen et al., 2018; Terwee et al., 2018). Firstly, Yoon et al., (2021a) assessed measures’ content validity for being the most important psychometric property when selecting a measure (Prinsen et al., 2016, 2018); if the content (e.g. items) of measures inadequately represents the construct(s) to be assessed, the evaluation of other psychometric properties is of limited value. This review by Yoon et al., (2021a) identified 15 parent- or caregiver-reported measures developed and published in English, assessed parents’ or caregivers’ attitude toward CM or perpetration of CM and assessed one or more of the four categories of CM (i.e. physical abuse, emotional abuse, sexual abuse and neglect; Slep et al., 2015; WHO, 2006, 1999). No high-quality evidence supporting insufficient content validity was found for any of the 15 included measures, thus rendering them suitable for further psychometric evaluation. In a subsequent psychometric review, Yoon et al., (2021b) reported on the other psychometric properties (reliabilities and validities other than content validity) of the 15 included measures (Mokkink, Prinsen, et al., 2018; Prinsen et al., 2018). However, responsiveness was outside the scope of this review by Yoon et al., (2021b), given that the search strategy needed to be adjusted to identify studies appropriate to determine responsiveness. No systematic review on the responsiveness of parent- or caregiver-reported measures on CM has been published to date.

### Study Aim

The aim of this systematic review was to evaluate responsiveness of all current parent- or caregiver-reported CM measures limited to one aspect of the construct approach for responsiveness (i.e. the comparison before and after interventions using the COSMIN guidelines; Mokkink, Prinsen, et al., 2018; Prinsen et al., 2018). Accordingly, the focus of this review is on which measures are sensitive enough to measure change over time in response to a parenting intervention (i.e. responsiveness of measures), not which interventions are effective (i.e. effectiveness of interventions). Due to the size, scope and complexity of reporting, the remaining aspects of the construct approach for responsiveness (i.e. comparison with other outcome measures and comparison between subgroups) were beyond the scope of the present review.

## Method

**Figure 1.**
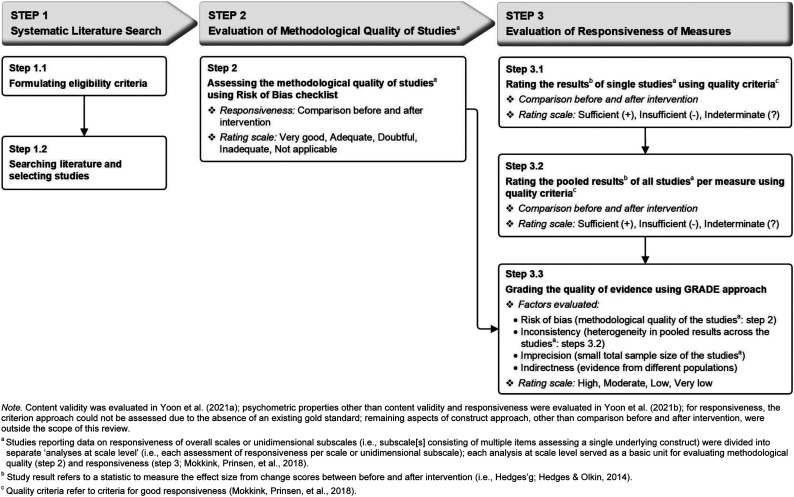
Study design: Steps for Preferred Reporting Items for Systematic Reviews and Meta-Analyses (Step 1) and COnsensus-based Standards for the selection of health Measurement INstruments processes (Steps 2 and 3).

This systematic review followed the guidelines of the Preferred Reporting Items for Systematic reviews and Meta-Analyses (PRISMA) statement (Moher et al., 2009) and the COSMIN guidelines (Prinsen et al., 2018). This review followed the following three consecutive steps (see Figure 1):

• Step 1: *Systematic literature search* formulating eligibility criteria (Step 1.1), searching the literature and selecting studies (Step 1.2);
• Step 2: *Evaluation of the methodological quality of studies* on responsiveness of measures using the COSMIN Risk of Bias checklist and
• Step 3: *Evaluation of responsiveness of measures* by rating the result of single studies against the criteria for responsiveness (Step 3.1), rating the pooled results of all studies per measure (Step 3.2) and grading the quality of evidence on responsiveness (Step 3.3).

Each of these steps will be described in more detail in the following sections.

### Step 1: Systematic Literature Search

The systematic literature search was performed formulating eligibility criteria (Step 1.1) and searching literature and selecting studies (Step 1.2) in accordance with the PRISMA statement (Moher et al., 2009). The PRISMA statement includes a 27-item checklist of elements deemed essential for conducting and reporting systematic reviews in a transparent manner. A completed PRISMA checklist for the current review is presented in Online Supplemental Table S1.

#### Eligibility Criteria (Step 1.1)

To be selected for this current review, articles had to meet the following three eligibility criteria: (1) journal articles were published in English; (2) articles involved parents or caregivers to assess their attitudes toward CM or change maltreating behaviours toward their children and (3) articles reported on responsiveness data (i.e. change scores of a measure before and after any intervention to reduce CM) for one or more of the 15 parent- or caregiver-reported CM measures (see Table 1 on the characteristics of the 15 identified measures) as identified in the companion systematic reviews by Yoon et al. (2021a, 2021b). In summary, any study that measured and compared parents’ or caregivers’ attitudes towards CM or maltreating behaviours towards their children before and after parenting interventions using any of the 15 identified measures were selected, regardless of their design.

**Table 1.** Characteristics of the Measures Assessing Child Maltreatment.

Measure (abbreviation)	Studies on development and validation	Main constructs	(Sub)scales	Target population	Purpose of use	No. of subscales (No. of items)	Range of score	Response options	Recall period	Cost (mode of administration)
Adult Adolescent Parenting Inventory-2 (AAPI-2)	Bavolek and Keene, 1999	Physical abuse; emotional abuse; neglect	Inappropriate parental expectations; Parental lack of an empathic awareness of children’s needs; strong belief in the use and value of corporal punishment; parent–child role reversal; oppressing children’s power and independence	Current andprospective parent populations	To identify maltreating parents/carers; to evaluate effectiveness of an intervention	5 (40)	0–50 (raw total scores per subscale are converted into standard scores: range 0–10)	5-point ordinal scale (strongly disagree = 1 to strongly disagree = 5)	Not specified	2 to 10 US dollars per administration (both paper- and web-based format)
Analog Parenting Task (APT)	Russa and Rodriguez, 2010; Zaidi et al. 1989	Physical abuse	Physical discipline; escalation of physical discipline	Prospective parent populations	To identify maltreating parents/carers	2 (26)	0–26	10 nominal scale (from nonphysical discipline tactics to physical discipline tactics)	Not specified	Freely available (computer-based format)
Child Neglect Questionnaire (CNQ)	Stewart et al., 2015	Neglect	Physical neglect; Emotional neglect; educational neglect; Supervision neglect	Parents with older children	To identify maltreating parents/carers	4 (46)	46–184	4-point ordinal scale (always = 1 to never = 4)	Past 6 months	Freely available (paper-based format)
Child Neglect Scales-Maternal Monitoring and Supervision Scale (CNS-MMS)	Kirisci et al., 2001	Neglect	Child neglect	Mothers	To evaluate effectiveness of an intervention	1 (11)	11–33	3-point ordinal scale (hardly ever = 1 to often = 3)	Past 6 months	Freely available (paper-based format)
Child Trauma Screen-Exposure Score (CTS-ES)	Lang and Connell, 2017	Physical abuse; emotional abuse; sexual abuse; neglect	Potentially traumatic event	Caregivers	To identify maltreating parents/carers	1 (4)	0–4	Dichotomous scale (no = 0 or yes = 1)	Not specified	Freely available (paper-based format)
Conflict Tactics Scales: Parent-Child version (CTSPC)	Straus et al., 1998; Straus et al., 2003	Physical abuse; emotional abuse	Nonviolent discipline; psychological aggression; physical assault	Parents	To identify maltreating parents/carers; to evaluate effectiveness of an intervention	3 (22)	0–550 (raw scores per item are converted into frequency scores: 0 = 0, 1 = 1, 2 = 2, 3–5 = 4, 6–10 = 8, 11–20 = 15 and > 20 = 25)	8-point ordinal scale ( 0 = never happened; 1 = once in the past year; 2 = twice; 3 = 3–5 times; 4 = 6–10 times; 5 = 11–20 times; 6 = more than 20 times; 7 = not in the past year, but it happened before)	Past 1 year	62 US dollars per pack of 25 questionnaires (paper-based format)
Family Maltreatment-Child Abuse criteria (FM-CA)	Heyman et al., 2020	Physical abuse; emotional abuse	Physical child abuse; psychological child abuse	Parents	To identify maltreating parents/carers; to evaluate effectiveness of an intervention	2 (27)	0–63	Dichotomous scale for physical child abuse subscale (I did = 0 or I never did = 1); 6-point ordinal scale for psychological child abuse subscale (never = 0 to more than once a day = 5)	Past 1 year	Freely available (computer-based format)
ISPCAN Child Abuse Screening Tool for use in Trials (ICAST-Trial)	Meinck et al., 2018	Physical abuse; emotional abuse; sexual abuse; neglect	Physical abuse; emotional abuse; contact sexual abuse; neglect	Caregivers	To evaluate effectiveness of an intervention	4 (14)	0-112	9-point ordinal scale (never = 0 to more than 8 times = 8)	Past 1 month	Freely available (both paper- and computer-based format)
Intensity of Parental Punishment Scale (IPPS)	Gordon et al., 1979	Physical abuse; emotional abuse	School misbehaviour; disobedience after a recent reminder; public disobedience; crying; destructiveness	Parents	To identify maltreating parents/carers; to evaluate effectiveness of an intervention	5 (33)	33–231	7-point ordinal scale (no reaction = 1 to very strong punishment = 7)	Not specified	Freely available (paper-based format)
Mother-Child Neglect Scale (MCNS)	Lounds et al., 2004	Neglect	Emotional neglect; cognitive neglect; supervisory neglect; physical needs neglect	Mothers	To identify maltreating parents/carers	4 (20)	20–80	4-point ordinal scale (strongly disagree = 1 to strongly agree = 4)	Past 1 year	Freely available (paper-based format)
Mother-Child Neglect Scale-Short Form (MCNS-SF)	Lounds et al., 2004	Neglect	Emotional neglect; cognitive neglect; supervisory neglect; Physical needs neglect	Mothers	To identify maltreating parents/carers	4 (8)	8–32	4-point ordinal scale (strongly disagree = 1 to strongly agree = 4)	Past 1 year	Freely available (paper-based format)
Parent-Child Aggression Acceptability Movie Task (P-CAAM)	Rodriguez et al., 2011	Physical abuse	Physical discipline; Physical abuse	Current and prospective parent populations	To identify maltreating parents/carers; to evaluate effectiveness of an intervention	2 (8 video clips: 90 sec each)	0–NR	Clips builds towards ‘initial physical contact between caregiver and child’; respondents should identify that moment and stop video; delay between actual physical contact and stop video = score (per video)	Not specified	Freely available (computer-based format)
Parent Opinion Questionnaire (POQ)	Azar and Rohrbeck, 1986	Physical abuse; emotional abuse; Neglect	Self-care; family responsibility and care of siblings; help and affection to parents; leaving children alone; proper behaviour and feelings; punishment	Parents	To identify maltreating parents/carers	6 (60)	0–60	Dichotomous scale (disagree = 0 or agree = 1)	Not specified	Freely available (paper-based format)
Parental Response to Child Misbehavior Questionnaire (PRCM)	Holden and Zambarano, 1992; Vittrup et al., 2006	Physical abuse; emotional abuse	Discipline techniques	Parents with young children	To identify maltreating parents/carers; to evaluate effectiveness of an intervention	1 (12)	0–72	6-point ordinal scale (never = 0 to 9 ≥ times per week = 6)	Past 1 week	Freely available (paper-based format)
Shaken Baby Syndrome Awareness Assessment-Short Version (SBS-SV)	Russell, 2010	Physical abuse; emotional abuse; neglect	Soothing techniques; discipline techniques; potential for injury	Parents and caregivers of young children	To evaluate effectiveness of an intervention	3 (36)	36–216	6-point ordinal scale (strongly disagree = 1 to strongly agree = 6)	Not specified	Freely available (paper-based format)

*Notes.* All information was derived from studies on development and validation of the measures.

NR = Not Reported.

#### Literature Search and Study Selection (Step 1.2)

To identify eligible articles that reported on responsiveness of the selected 15 measures, systematic literature searches were performed in six electronic databases: CINAHL, Embase, ERIC, PsycINFO, PubMed and Sociological Abstracts. All database searches were conducted in January 2020 with an updated search conducted in March 2021. Free text terms were used to search databases and to retrieve all publication prior to March 2021 (see Online Supplemental Table S2 for the search strategies for the current review).

Titles and abstracts retrieved from database searches were screened to identify eligible journal articles on responsiveness of the 15 measures by two reviewers independently; one reviewer screened all abstracts, while the other reviewer screened a random selection of 50% of all abstracts. All full texts of eligible abstracts were retrieved and assessed by both reviewers independently. Any disagreements between both reviewers were resolved via a consensus decision including a third reviewer. Inter-rater agreement was determined using Cohen’s weighted κ (Cohen & Humphreys, 1968) and interpreted as: very good (**κ** = 0.81–1.00), good (κ = 0.61–0.80), moderate (κ = 0.41–0.60), fair (κ = 0.21–0.40) and poor (κ = 0.00–0.20) agreement (Altman, 1991). Reference lists of all included full-text articles were searched manually to identify additional eligible journal articles. Hand searching of reference lists was performed by one reviewer and identified journal articles were checked by the second reviewer.

After identifying eligible articles, a distinction was made between ‘an article’ and ‘an analysis at scale ‘level.’ An article may assess responsiveness of (a) one overall scale or (b) one overall scale and several unidimensional subscales (i.e. subscale(s) consisting of multiple items that assess a single underlying construct) or (c) several unidimensional subscales. Conversely, an analysis at scale level assess only one overall scale or one unidimensional subscale, thus making it the lowest unit of analysis to determine responsiveness (Mokkink, Prinsen, et al., 2018). This is an important distinction as authors report on the effectiveness of interventions using both overall scales and subscales – hence the need to assess responsiveness of both all overall scales as well as unidimensional subscales. The unidimensionality of a subscale was confirmed if data could be identified in the literature supporting the internal structure of the subscale (i.e. conducted factor analysis and internal consistency using Cronbach’s alpha for each subscale; Mokkink, de Vet, et al., 2018). The confirmed subscale can be used as an independent measure besides an overall scale (Mokkink, Prinsen, et al., 2018). Included articles reporting data on responsiveness of overall scales or confirmed subscales were divided into separate ‘analyses at scale level’ (i.e. each assessment of responsiveness per scale or unidimensional subscale) for evaluation of methodological quality of studies (Step 2). When relevant data on responsiveness were not available from the included articles, the authors were contacted for additional information.

### Step 2: Evaluation of Methodological Quality of Studies

The methodological quality of the included studies on the responsiveness of the selected 15 measures was assessed using the COSMIN Risk of Bias checklist (Mokkink, de Vet, et al., 2018). The checklist contains three items for responsiveness on comparison *before and after intervention* (see Online Supplemental Table S3), which rate the quality of study design and the robustness of statistical methods used in studies on a measure’s responsiveness to change following intervention (Mokkink, de Vet, et al., 2018). Each checklist item was scored on a four-point rating scale: *inadequate* = 1, *doubtful* = 2, *adequate* = 3 and *very good* = 4 (Mokkink, de Vet, et al., 2018). A total rating for responsiveness was determined by the ratio of *‘the obtained total score minus the minimum possible score’* to *‘the maximum possible score minus the minimum possible score’* (Cordier et al., 2015). This ratio score method was preferred over the worst score counts method as suggested by the COSMIN guidelines (i.e. determining total ratings based on the lowest rating of any of the checklist items; Mokkink, Prinsen, et al., 2018). The worst score counts method is likely to prohibit detecting subtle differences in methodological quality between studies (Speyer et al., 2014). Accordingly, the total score of methodological quality ratings on responsiveness was reported as a percentage rating and can be interpreted as follows: inadequate (from 0% to 25%), doubtful (from 25.1% to 50%), adequate (from 50.1% to 75%) and very good (from 75.1% to 100%). Two independent reviewers rated the methodological quality. Any disagreements were resolved by consensus. The inter-rater agreement between both reviewers was determined by weighted κ (Cohen & Humphreys, 1968). Unpublished literatures were excluded due to the recognised difficulties in systematically searching them when there is a lack of registries for relevant studies (Egger et al., 2003) and their tendency towards low methodological quality (Conn et al., 2003).

After assessing methodological quality of the included studies on responsiveness, the following data from the included studies and measures were extracted using a data extraction template that is part of the COSMIN manual (Mokkink, Prinsen, et al., 2018): (1) study characteristics; (2) measure characteristics and (3) study results on responsiveness. (i.e. conducted factor analysis and internal consistency using Cronbach’s alpha for each subscale; Mokkink, de Vet, et al., 2018) The extraction was done by one reviewer and a second reviewer cross-checked the accuracy and completeness of the extracted data. All extracted data were used for evaluation of responsiveness of measures (Step 3).

### Step 3: Evaluation of Responsiveness of Measures

The responsiveness of measures was assessed in three sequential steps: Step 3.1 rating the results of single studies, Step 3.2 rating the pooled results of all studies per measure and Step 3.3 grading the quality of evidence on responsiveness. All ratings were scored by two independent reviewers separately, after which consensus ratings were determined based on reviewers’ group discussion.

#### Rating the Results of Single Studies (Step 3.1)

Rating the results of single studies using quality criteria for responsiveness was limited to the comparison of *before and after intervention*. The results of responsiveness to change in scores following an intervention for each individual study were rated as *sufficient* (+ = meeting the quality criteria), *insufficient* (− = below the quality criteria) or *indeterminate* (? = lack of robust evidence of meeting the quality criteria) against predefined criteria for good responsiveness (Mokkink, Prinsen, et al., 2018; see Online Supplemental Table S4). For a sufficient (+) rating on single study results, robust data on change scores before and after intervention on the selected measures should be available to allow calculation of the standardised mean difference (SMD) and confirm at least medium effect size (i.e. Hedges’ *g* ≥ 0.50; Cohen, 1988); insufficient (−) ratings showed calculated SMDs below medium effect size (i.e. Hedges’ *g* < 0.50; Cohen, 1988). Single study results that did not provide robust data to allow SMD calculations (Hedges’ *g*; Hedges & Olkin, 2014) were rated as indeterminate (?). Although the SMD is conventionally estimated by Cohen’s *d*, Cohen’s *d* tends to overestimate the SMD when the sample size is small (Cohen, 1988). As the small sample bias of Cohen’s *d* can be corrected by Hedges’ *g*, the SMD was estimated with Hedges’ *g* in this review to reflect the most accurate estimate of effect sizes (Cohen, 1988).

#### Rating the Pooled Results of All Studies Per Measure (Step 3.2)

All results on responsiveness from available studies per measure were quantitatively pooled into overall ratings of the responsiveness per measure (Prinsen et al., 2018). An overall sufficient (+), insufficient (−) or indeterminate (?) rating for responsiveness was given using the same quality criteria for good responsiveness (Mokkink, Prinsen, et al., 2018) (see Online Supplemental Table S4). For an overall sufficient (+) rating on responsiveness per measure, the pooled SMD must be at least medium effect size (i.e. Hedges’ *g* ≥ 0.50; Cohen, 1988). For an overall insufficient (−) rating, the pooled SMD falls below medium effect size (i.e. Hedges’ *g* < 0.50; Cohen, 1988). For an overall indeterminate (?) rating, all results represent insufficiently robust data, thus not supporting the calculation of the pooled SMD (Hedges’ *g*; Hedges & Olkin, 2014). Hedges’ *g* for both single study results (Step 3.1) and all study results per measure (Step 3.2) was calculated as proposed by Borenstein et al. (2009) and using the Comprehensive Meta-Analysis (CMA) software version 3.0 (Borenstein et al., 2013). In cases where at least moderate heterogeneity (i.e. Higgins’ *I*^*2*^ ≥ 50%; Higgins et al., 2003) in effect sizes across studies were calculated (Higgins et al., 2003), a random effect model (Borenstein et al., 2009) was used to calculate pooled effect size. In cases where low heterogeneity (i.e. 0 ≤ *I*^*2*^ < 50%; Higgins et al., 2003) was calculated, a fixed effect model was used by giving relatively greater weight to individual studies with larger sample sizes in contrast to the random effect model that does not take into account the weight of samples sizes when calculating pooled effect size (Borenstein et al., 2009).

To assess the impact of publication bias (i.e. the tendency for studies reporting large and significant intervention effects to be published more commonly than those with small and non-significant effects based on small sample size, Higgins & Green, 2011) on the pooled effect size, two consecutive tests were performed using the CMA software 3.0 (Borenstein et al., 2013). The Begg and Muzumdar’s rank correlation test (Begg’s test) was first performed to exam the relationship between the standardised effect size and the sample size across studies (Begg & Mazumdar, 1994). A Begg’s test two-tailed *p*-value of less than 0.05 indicated significant publication bias existed in the pooled effect size (Begg & Mazumdar, 1994). This significant bias may inflate the pooled effects as small sample studies with small effects are potentially unpublished and missing (Begg & Mazumdar, 1994). If the publication bias existed, trim-and-fill test by Duval and Tweedie (2000) was next performed using the fixed effect model to examine how much impact the missing unpublished studies have had on the pooled effect size. The test investigates the publication bias funnel plot in which the individual effect size from each study is plotted relative to the study’s standard error (Duval & Tweedie, 2000). The plot is expected to be symmetric, which means studies will be distributed equally on either side of the pooled effect (Higgins & Green, 2011). The trim-and-fill test corrects publication bias by filling the funnel plot with studies that were potentially missing to make the funnel plot symmetric (Duval & Tweedie, 2000). The trim-and-fill test also produces an adjusted pooled effect size and confidence interval after accounting for missing studies due to publication bias (Duval & Tweedie, 2000). The publication bias was not tested when there were less than three studies (Higgins & Green, 2011).

#### Grading the Quality of Evidence on Responsiveness (Step 3.3)

The quality of the evidence (i.e. the entire body of evidence used for overall ratings on responsiveness per measure) was graded as *high*, *moderate*, *low* and *very low* evidence, using a modified Grading of Recommendations Assessment, Development and Evaluation (GRADE) approach (Mokkink, Prinsen, et al., 2018; see Online Supplemental Table S5). The modified GRADE approach assumes that the initial quality of evidence used for overall ratings is of high-quality. Subsequently, the quality of evidence is downgraded by one-to-three levels (to moderate, low or very low) when there are serious (−1: one level down), very serious (−2: two levels down) or extremely serious (−3: three levels down) concerns across the evidence. The quality ratings of evidence were determined taking into consideration the following four factors: (a) risk of bias (limitations in the methodological quality of studies (Step 2); (b) inconsistency (heterogeneity in pooled results of studies (Step 3.2); (c) indirectness (evidence from different populations other than the target population in the review) and (d) imprecision (a low total sample size included in the studies) (Mokkink, Prinsen, et al., 2018). Quality of evidence should not be graded if the overall rating was indeterminate (?) due to lack of robust evidence (Prinsen et al., 2018). Publication bias was not considered in the modified GRADE approach due to a lack of registries for studies on psychometric properties according to the COSMIN manual (Mokkink, Prinsen, et al., 2018). More detailed information on grading quality of evidence can be found in the COSMIN manual for systematic reviews of measures (Mokkink, Prinsen, et al., 2018).

## Results

### Systematic Literature Searches (Step 1)

**Figure 2.**
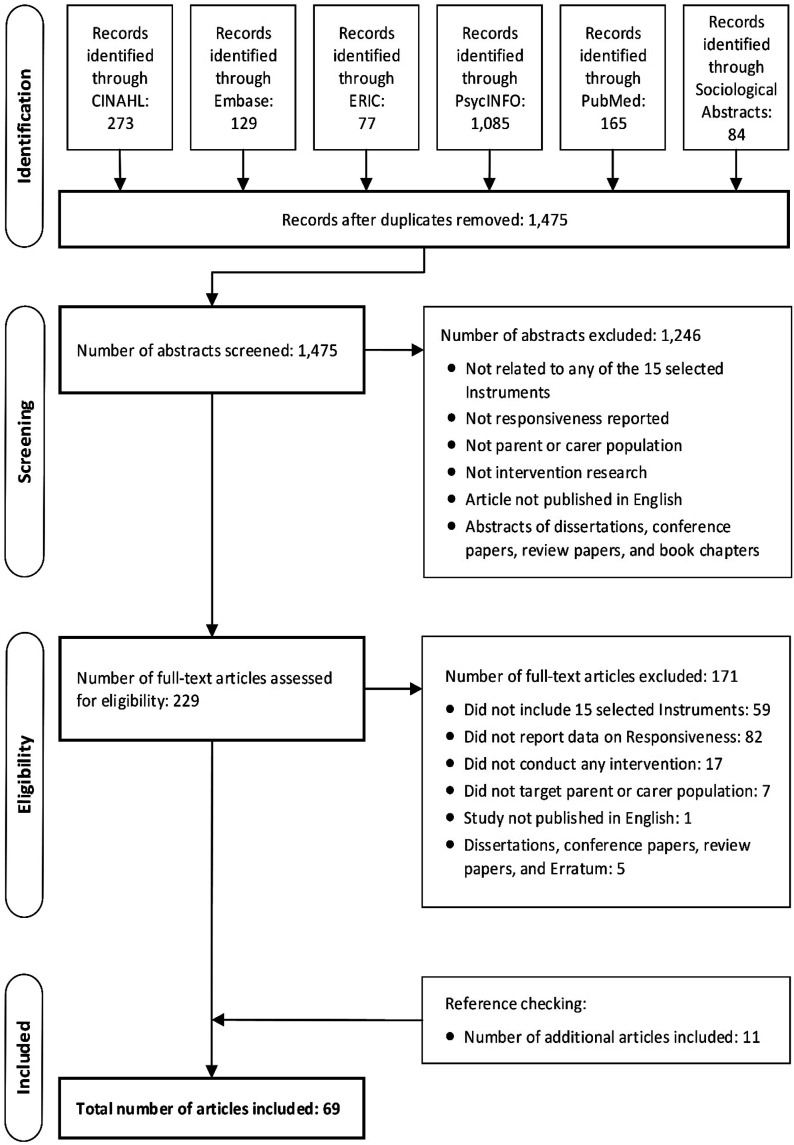
Flow diagram of the reviewing procedure based on Preferred Reporting Items for Systematic Reviews and Meta-Analyses (Moher et al., 2009).

A total of 1475 abstracts were identified from six electronic databases after removing duplicates: 273 records in CINAHL; 129 records in Embase; 77 records in ERIC; 1085 records in PsycINFO; 165 records in PubMed and 84 records in Sociological Abstracts. Figure 2 shows the flow chart of the studies identified during literature searching and study selection (Step 1.2) in accordance with PRISMA (Moher et al., 2009). A total of 229 full-text articles were assessed for eligibility, of which 58 journal articles met all inclusion criteria: 171 articles did not meet at least one of the inclusion criteria. Reference checking of the included 58 journal articles identified 11 additional articles meeting all inclusion criteria. As a result, 69 journal articles reporting on the responsiveness of 15 parent- or caregiver-reported CM measures were included in this review. General characteristics of the included 69 articles are presented in Online Supplemental Table S6. Furthermore, as most included articles presented data on the responsiveness of more than one overall scale or unidimensional subscale, the included 69 articles contained 223 analyses at scale level for the quality assessment of the study (Step 2) and the responsiveness (Step 3). The inter-rater agreement for selection of articles between two reviewers was very good (Altman, 1991): weighted κ for abstract selection = 0.81 (95% confidence interval [CI] = [0.74, 0.88]); weighted κ for article selection = 0.83 (95% CI [0.75, 0.90]).

### Methodological Quality of the Included Studies (Step 2)

The methodological quality of the 223 analyses at scale level in 69 included articles on responsiveness was assessed using the COSMIN Risk of Bias checklist (Mokkink, de Vet, et al., 2018). Table 2 presents an overview of all methodological quality ratings for the 223 analyses at scale level on responsiveness of 15 measures. In total, 57% (127/223) of analyses at scale level reporting on responsiveness were scored as having good or adequate methodological quality, whereas 43% (96/223) were scored as having doubtful or inadequate quality. The inter-rater agreement for study quality assessment between both reviewers was very good: weighted κ = 0.83 (95% CI [0.77, 0.91]).

**Table 2.** Methodological Quality Assessment on Responsiveness of Measures: Summary of Findings for Step 2 in Figure 1.

Measures	Overall scale / subscale^a^	Number of analyses at scale level on methodological quality^b^			
		Very good	Adequate	Doubtful	Inadequate
AAPI-2	Overall scale	13	10	16	4
	Inappropriate Expectations subscale	7	5	13	2
	Lack of Empathy subscale	8	6	13	2
	Oppressing Children’s Power and Independence subscale	6	4	12	2
	Role Reversal subscale	6	6	13	2
	Value of Corporal Punishment subscale	7	6	11	2
APT	Overall scale	1	0	0	0
CNQ	Overall scale	NR			
CNS-MMS	Overall scale	NR			
CTS-ES	Overall scale	NR			
CTSPC	Overall scale	8	7	1	0
	Physical Assault subscale	6	4	0	0
FM-CA	Overall scale	0	1	0	0
ICAST-Trial	Overall scale	2	1	1	0
	Emotional Abuse subscale	1	1	0	0
	Neglect subscale	2	1	1	0
	Physical Abuse subscale	1	1	0	0
	Sexual Abuse subscale	1	1	0	0
IPPS	Overall scale	NR			
MCNS	Overall scale	1	0	0	0
MCNS-SF	Overall scale	NR			
P-CAAM	Overall scale	NR			
POQ	Overall scale	1	1	0	0
PRCM	Overall scale	1	0	1	0
SBS-SV	Overall scale	NR			

Abbreviation: AAPI-2 = Adult Adolescent Parenting Inventory-2; APT = Analog Parenting Task; CNQ = Child Neglect Questionnaire; CNS-MMS = Child Neglect Scales-Maternal Monitoring and Supervision Scale; CTS-ES = Child Trauma Screen-Exposure Score; CTSPC = Conflict Tactics Scales: Parent-Child version; FM-CA = Family Maltreatment-Child Abuse criteria; ICAST-Trial = ISPCAN (International Society for the Prevention of Child Abuse and Neglect) Child Abuse Screening Tool for use in Trials; IPPS = Intensity of Parental Punishment Scale; MCNS = Mother-Child Neglect Scale; MCNS-SF = Mother-Child Neglect Scale-Short Form; P-CAAM = Parent-Child Aggression Acceptability Movie task; POQ = Parent Opinion Questionnaire; PRCM = Parental Response to Child Misbehavior questionnaire; SBS-SV = Shaken Baby Syndrome awareness assessment-Short Version.

^a^Subscales were included if data on factor analysis and Cronbach’s alpha determined per subscale could be retrieved from the literature, thus confirming the scale’s multidimensional structure (Mokkink, Prinsen, et al., 2018).

^b^The methodological quality was rated using the COSMIN checklist (Mokkink, de Vet, et al., 2018): very good, adequate, doubtful, inadequate and NR (not reported); detailed rating results on methodological quality of single studies can be founded in Online Supplemental Table S7.

### Responsiveness and Quality of Evidence of Measures (Step 3)

Table 3 summarises ratings on responsiveness for analyses at scale level (Step 3.1); the results of analyses at scale level and their quality ratings are presented in detail in Online Supplemental Table S7. Online Supplemental Table S7 shows that the results of scale level analyses with inadequate methodological quality tend to have smaller effect sizes than analyses with better methodological quality. Although the results from inadequate analyses might be biased, these results were included when pooling the results (Step 3.2) from all analyses per CM measure, because the pooled results should be considered to downgrade the quality of the evidence (Step 3.3) in terms of their risk of bias (Mokkink, Prinsen, et al., 2018). All extracted data on responsiveness from the 223 analyses at scale level (from 69 included articles) were evaluated against the criteria for good responsiveness (Prinsen et al., 2018; see Online Supplemental Table S4). Of all 223 ratings on responsiveness data of analyses at scale level, only four ratings received an indeterminate rating due to less robust data being reported on responsiveness (see Table 3). All other analyses at scale level results received either a sufficient (69/223) or an insufficient (150/223) rating on responsiveness.

**Table 3.** Ratings of Single Analysis at Scale Level Results on Responsiveness: Summary of Findings for Step 3.1 in Figure 1.

Measure	Overall scale/subscale^a^	Number of each rating on single scale analysis results on responsiveness^b^		
		+	−	?
AAPI-2	Overall scale	12	29	2
	Inappropriate Expectations subscale	5	22	0
	Lack of Empathy subscale	13	16	0
	Oppressing Children’s Power and Independence subscale	5	19	0
	Role Reversal subscale	8	19	0
	Value of Corporal Punishment subscale	9	17	0
APT	Overall scale	1	0	0
CNQ	Overall scale	NR		
CNS-MMS	Overall scale	NR		
CTS-ES	Overall scale	NR		
CTSPC	Overall scale	5	9	2
	Physical Assault subscale	4	6	0
FM-CA	Overall scale	1	0	0
ICAST-Trial	Overall scale	1	3	0
	Emotional Abuse subscale	0	2	0
	Neglect subscale	0	4	0
	Physical Abuse subscale	2	0	0
	Sexual Abuse subscale	0	2	0
IPPS	Overall scale	NR		
MCNS	Overall scale	0	1	0
MCNS-SF	Overall scale	NR		
P-CAAM	Overall scale	NR		
POQ	Overall scale	2	0	0
PRCM	Overall scale	1	1	0
SBS-SV	Overall scale	NR		

Abbreviation: AAPI-2 = Adult Adolescent Parenting Inventory-2; APT = Analog Parenting Task; CNQ = Child Neglect Questionnaire; CNS-MMS = Child Neglect Scales-Maternal Monitoring and Supervision Scale; CTS-ES = Child Trauma Screen-Exposure Score; CTSPC = Conflict Tactics Scales: Parent-Child version; FM-CA = Family Maltreatment-Child Abuse criteria; ICAST-Trial = ISPCAN (International Society for the Prevention of Child Abuse and Neglect) Child Abuse Screening Tool for use in Trials; IPPS = Intensity of Parental Punishment Scale; MCNS = Mother-Child Neglect Scale; MCNS-SF = Mother-Child Neglect Scale-Short Form; P-CAAM = Parent-Child Aggression Acceptability Movie task; POQ = Parent Opinion Questionnaire; PRCM = Parental Response to Child Misbehavior questionnaire; SBS-SV = Shaken Baby Syndrome awareness assessment-Short Version; NR = not reported.

^a^Subscales were included if data on factor analysis and Cronbach’s alpha determined per subscale could be retrieved from the literature, thus confirming the scale’s multidimensional structure (Mokkink, Prinsen, et al., 2018).

^b^The single analysis at scale level results on responsiveness was rated in Step 3 of Figure 1, using the criteria for good responsiveness (Mokkink, Prinsen, et al., 2018): + = sufficient, − = insufficient, ? = indeterminate (due to less robust psychometric data) and NR = not reported (due to no data on responsiveness); detailed single analysis at scale level results and ratings on each responsiveness are available in Online Supplemental Table S7.

Table 4 summarises the overall responsiveness ratings (Step 3.2) and the quality of evidence (Step 3.3) for responsiveness per overall scale or subscale of all 15 measures. The pooled results of all analyses at scale level on responsiveness for each overall scale or subscale and detailed reasons for downgrading on quality of all evidence used for the overall ratings are displayed in Online Supplemental Table S8. The overall rating for pooled results of analyses at scale level on responsiveness for each overall scale or subscale were evaluated using the same criteria for good responsiveness (Prinsen et al., 2018; see Online Supplemental Table S4). None of the overall scales and subscales for the 15 measures received an indeterminate overall rating for responsiveness (see Table 4). Almost half of all measures (7 out of 15) received ‘not reported’ (NR) as overall ratings because no data on responsiveness could be retrieved from the included studies. Of the remaining 8 measures, only three measures and one subscale received an overall sufficient responsiveness; all the others received an overall insufficient rating on responsiveness. The publication bias *p*-value by Begg’s test and adjusted pooled effect sizes after correcting the publication bias by using the trim-and-fill test are presented in Online Supplemental Table S8. Although one subscale was affected by significant publication bias (i.e. Begg’s test *p*-value <0.05), the subscale’s overall responsiveness rating on the adjusted pooled result after accounting for the publication bias was not changed compared with the overall rating on the unadjusted result as the adjusted pooled effect size was also smaller than the medium effect size (i.e. Hedges’ *g* < 0.5) and the same as the unadjusted size. None of the other pooled effect sizes were significantly affected by publication bias (i.e. Begg’s test *p*-value ≥0.05). In addition, the quality of evidence (confidence level for the overall rating per overall scale or subscale) was evaluated using the modified GRADE approach (Prinsen et al., 2018; see Online Supplemental Table S5). Again, measures (7 out of 15) that had not reported on responsiveness data, received ‘not reported’ (NR) as quality ratings of evidence (see Table 4). Of the remaining 8 measures, only one single subscale reported a high-quality evidence supporting its overall rating on responsiveness; all the others reported either moderate or low-quality evidence for their overall ratings on responsiveness.

**Table 4.** Overall Ratings on Pooled Study Results and Quality of Evidence on Responsiveness Per Measure: Summary of Findings for Steps 3.2 and 3.3 in Figure 1.

Measure	Overall scale/subscale^a^	Overall rating^b^	Quality of evidence^c^
AAPI-2	Overall scale	−	Low
	Inappropriate Expectations subscale	−	Low
	Lack of Empathy subscale	−	Low
	Oppressing Children’s Power and Independence subscale	−	Low
	Role Reversal subscale	−	Low
	Value of Corporal Punishment subscale	−	Low
APT	Overall scale	+	Low
CNQ	Overall scale	NR	NR
CNS-MMS	Overall scale	NR	NR
CTS-ES	Overall scale	NR	NR
CTSPC	Overall scale	−	Low
	Physical Assault subscale	−	Low
FM-CA	Overall scale	+	Moderate
ICAST-Trial	Overall scale	−	Low
	Emotional Abuse subscale	−	Low
	Neglect subscale	−	Low
	Physical Abuse subscale	+	High
	Sexual Abuse subscale	−	Moderate
IPPS	Overall scale	NR	NR
MCNS	Overall scale	−	Moderate
MCNS-SF	Overall scale	NR	NR
P-CAAM	Overall scale	NR	NR
POQ	Overall scale	+	Moderate
PRCM	Overall scale	−	Moderate
SBS-SV	Overall scale	NR	NR

Abbreviation: AAPI-2 = Adult Adolescent Parenting Inventory-2; APT = Analog Parenting Task; CNQ = Child Neglect Questionnaire; CNS-MMS = Child Neglect Scales-Maternal Monitoring and Supervision Scale; CTS-ES = Child Trauma Screen-Exposure Score; CTSPC = Conflict Tactics Scales: Parent-Child version; FM-CA = Family Maltreatment-Child Abuse criteria; ICAST-Trial = ISPCAN (International Society for the Prevention of Child Abuse and Neglect) Child Abuse Screening Tool for use in Trials; IPPS = Intensity of Parental Punishment Scale; MCNS = Mother-Child Neglect Scale; MCNS-SF = Mother-Child Neglect Scale-Short Form; P-CAAM = Parent-Child Aggression Acceptability Movie task; POQ = Parent Opinion Questionnaire; PRCM = Parental Response to Child Misbehavior questionnaire; SBS-SV = Shaken Baby Syndrome awareness assessment-Short Version.

^a^Subscales were included if data on factor analysis and Cronbach’s alpha determined per subscale could be retrieved from the literature, thus confirming the scale’s multidimensional structure (Mokkink, Prinsen, et al., 2018).

^b^Overall ratings of pooled study results on responsiveness was rated in Step 3.2 of Figure 1, using the criteria for good responsiveness (Mokkink, Prinsen, et al., 2018); + = Sufficient rating, − = Insufficient rating and NR = not reported (due to no data on responsiveness); if the overall rating of a measure is sufficient, the measure is considered to be sufficiently responsive or sensitive to detect effects of interventions; detailed pooled results on responsiveness per measure are available in Online Supplemental Table S8.

^c^Level of quality of evidence (i.e. a degree of confidence on overall rating of responsiveness) was graded in Step 3.3 of Figure 1, using the modified GRADE approach for grading the quality of summarised evidence on responsiveness (Mokkink, Prinsen, et al., 2018): High = high level of confidence, Moderate = moderate level of confidence, Low = low level of confidence, Very Low = very low level of confidence, NR = not reported (due to not reported overall rating of responsiveness); if the evidence quality is very low, we should be concerned about using the overall ratings alone to recommend good measures; reasons for each grading on quality of evidence are available in Online Supplemental Table S8.

## Discussion

The aim of this systematic review was to evaluate quality of responsiveness (comparison before and after interventions) of all current parent- or caregiver-reported measures on CM by parents or caregivers using the recently revised COSMIN guidelines. This review identified 69 articles that reported on responsiveness of the 15 parent- or caregiver-reported CM measures identified by Yoon et al. (2021a, 2021b). The identified individual articles contained 223 analyses at scale level for each overall scale and subscale of the 15 measures. The methodological quality of the included studies was generally adequate. However, responsiveness data were only retrieved from the literature for about half of the included measures (8/15). Moreover, there is lack of high-quality evidence to support that the responsiveness of the measures is either sufficient or insufficient to determine the effect of parenting interventions for preventing CM. Only one subscale (ICAST-Trial [physical abuse]) reported high-quality evidence that it is sufficiently responsive to change before and after intervention. Due to lack of high-quality evidence on the responsiveness of overall scales and subscales, all of the measures included in this review may still have the potential to be used in interventions. However, additional robust research focusing on their responsiveness is needed before these measures can be recommended for use to determine the effectiveness of interventions (before and after measurement).

### Methodological Quality of the Included Studies

In terms of quality of study design, most of analyses at scale level (81 of 96) reporting doubtful or inadequate methodological quality (see Online Supplemental Table S7), as they had a methodological shortcoming (i.e. most studies were not designed as randomised controlled trials [RCTs]). As RCT randomly allocates study samples either to an intervention or a control group, it can minimise selection bias and confounding variables such as different sample characteristics (Altman, 1991). For this reason, RCT is considered to be the most powerful study design to estimate unbiased effect size of an intervention (Altman, 1991). However, only few RCTs have been conducted on the effectiveness of interventions to prevent CM due to practical issues related to cost effectiveness and ethical issues related to this socially sensitive research topic (van der Put et al., 2018). For this reason, if only RCT studies were to be included in this review, much data on responsiveness of parent- or caregiver-reported CM measures would have been excluded. This reasoning is also in line with a meta-analysis carried out by Gubbels et al. (2019), which noted that RCTs are rare in the field of CM. Thus, although many analyses at scale level showed poor methodological quality due to shortcomings in their study designs, no limitations to study design were applied in this review when retrieving data on responsiveness from the literature.

In terms of robustness of statistical methods, most of the analyses at scale level (78 of 96) were rated as having doubtful or inadequate methodological quality because they used a less robust statistical analysis, such as a paired *t*-test or a repeated-measures analysis of variance (ANOVA) reporting only *p*-values (see Online Supplemental Table S7). The *p*-value is an inappropriate measure of responsiveness (Mokkink, de Vet, et al., 2018) for the following two reasons: (1) it is only a statistic to confirm whether the estimated mean difference in scores before and after an intervention is likely not caused by chance (i.e. statistical significance) and it does not reflect whether the magnitude of the estimated mean difference is large enough to detect a clinically important effect (i.e. clinical significance) and (2) it is dependent on sample size (Altman, 1991). To account for these limitations of a *p*-value, an effect size (e.g. Hedges’ *g*, Hedges & Olkin, 2014) is preferred as an indicator of responsiveness in the COSMIN risk of bias checklist (Mokkink, Prinsen, et al., 2018), as it reflects the magnitude of mean difference before and after an intervention, regardless of sample sizes (Altman, 1991). However, most analyses at scale level only reported on *p*-values of paired *t*-tests or repeated-measures ANOVAs, resulting in doubtful or inadequate methodological study quality ratings.

For subscales, the methodological quality of studies was reported in only three out of eight measures reporting data on their responsiveness (Adult Adolescent Parenting Inventory-2 [AAPI-2], Conflict Tactics Scales: Parent-Child version [CTSPC] and ICAST-Trial). For the remaining five measures (Analog Parenting Task [APT], Family Maltreatment-Child Abuse criteria [FM-CA], Mother-Child Neglect Scale [MCNS], Parent Opinion Questionnaire [POQ] and Parental Response to Child Misbehavior questionnaire [PRCM]), the methodological quality of their subscales was not rated as the internal structure of their subscales was unclear and not confirmed by statistical analyses (i.e. by conducting statistical analysis to determine the factor structure and internal consistency). If a subscale has an unclear internal structure and unidimensionality cannot be confirmed (i.e. all items assess one underlying construct), then the construct of the subscale’s responsiveness has no further value (Prinsen et al., 2016), regardless of whether or not the subscale can detect treatment effects following intervention. For example, when a subscale on parental neglect also contains items that assess sexual abuse, the subscale would be of no use for capturing changes in parental neglect as different constructs are combined within the same subscale. However, most parent- or caregiver-reported CM measures has not been tested to confirm the internal structure of their subscales Yoon et al., (2021b), which could lead to either underestimating or overestimating the effectiveness of CM interventions (Meinck et al., 2018).

### Responsiveness of Measures

In general, evidence on responsiveness of a total of 25 overall scales or subscales was rated as either *sufficient* (3 overall scales and 1 subscale), *not reported* (7 overall scales) or *insufficient* (5 overall scales or 9 subscales). Insufficient responsiveness was due to not meeting the minimum criterion for good responsiveness (i.e. estimated effect size smaller than medium; Cohen, 1988). This review is based on current evidence on responsiveness as retrieved from the literature. Due to overall low quality of evidence of data, the estimated small effect sizes as presented in this review may change if future intervention studies provide high-quality evidence (Mokkink, Prinsen, et al., 2018). Therefore, the 14 measures for which no high-quality evidence could be identified may still have potential to be used for detecting changes in parental maltreating behaviours towards their children after intervention, if high-quality evidence are provided to support their responsiveness in future studies. Another important consideration in relation to the overall low to medium effect sizes is the quality of interventions. The findings suggest that new approaches to parent focussed CM interventions need to be considered to improve outcomes for both children and parents, which are more effective in changing parental attitude toward CM and reducing maltreating behaviours toward their children. For three overall scales (APT, FM-CA and POQ) and one subscale (ICAST-Trial [physical Abuse]), evidence on responsiveness was sufficient with estimated effect sizes higher than medium (Cohen, 1988). However, as quality of evidence for sufficient responsiveness of all three overall scales were rated as either moderate or low, the three overall scales need more robust evidence to be recommended for use in CM intervention. Only one single subscale (ICAST-Trial [Physical Abuse]) demonstrated high-quality evidence for responsiveness. Therefore, considering the most robust current evidence supporting sufficient responsiveness, only the Physical Abuse subscale of ICAST-Trial can be recommended as the most suitable measure for use in parenting interventions for reducing CM by parents.

Overall quality of evidence to support the responsiveness of parent- or caregiver-reported measures on CM was weak with mainly moderate to low ratings. The low quality of evidence was due to very inconsistent results across studies (i.e. substantial heterogeneity in the pooled effect sizes of studies). This substantial heterogeneity is in line with the previous meta-analysis on effects of parenting interventions to prevent CM by Chen and Chan (2016). The authors found a wide variation of effect sizes within groups of studies using the same measures on CM and between individual studies regardless of measures. Examining the influence of moderator variables on the heterogeneity, Chen and Chan (2016) found that characteristics of both sample (e.g. country income level and gender) and intervention (e.g. dosage and timing) contribute to significant between-study variance. However, there is no research, including Chen and Chan (2016), that focused on what variables contribute to the heterogeneity of effect sizes across studies on parenting interventions per parent- or caregiver-reported CM measure. Also, additional reasons for the poor evidence quality were small total sample sizes included in the studies (e.g. APT [*n* < 50] and POQ [*n* < 100]) and poor methodological quality of studies (e.g. FM-CA [only one study of adequate quality available]). Therefore, the quality of evidence to support the responsiveness of included measures was overall low due to concerns on inconsistent results across studies, small sample sizes and poor study quality.

The responsiveness of the AAPI-2 and the CTSPC, the two most widely used measures to assess the effectiveness of parenting interventions to prevent CM, was rated as overall insufficient with low quality of evidence. As such, they could not be recommended for use in parenting interventions to reduce CM. The frequent use of CM measures with low quality evidence can hamper the use of evidence-based parenting interventions. This issue may be the result of many clinicians tending to use measures based on the measures’ popularity in most clinical practices, rather than the quality of the measure’s responsiveness (Meinck et al., 2016, 2018). Therefore, selecting and using CM measures only based on its popularity rather than the psychometric evidence can lead to either the underestimation or the overestimation of a parenting intervention’s effectiveness which, in turn, can lead to the use of ineffective parenting interventions for preventing CM.

### Limitations

This systematic review has some limitations. Firstly, only measures developed in English and studies published in English were included. Accordingly, some findings on responsiveness of CM measures published in languages other than English may have been missed. Secondly, despite concerted efforts to contact authors for missing data, approximately 2% of data across the analyses at scale level were not retrieve (4 of 223, see Online Supplemental Table S7), which is negligible. Hence, the results on responsiveness of these four analyses at scale level were rated as indeterminate (Step 3.1) and were not pooled into overall ratings on responsiveness per measure (Step 3.2). Thirdly, publication bias could only be tested for three measures (AAPI-2, CTSPC and ICAST-Trial), as the remaining measures did not have the minimum number of studies required to allow the test. The Lack of Empathy subscale of AAPI-2 had the potential for publication bias with a significant Begg’s test result (*p* < 0.05). Fourthly, this review reported only on one aspect of the construct approach for responsiveness (comparison before and after intervention; Mokkink et al., 2010b). The other two aspects (i.e. comparison with other outcome measures and comparison between subgroups) were beyond the scope of the present review. To include these two aspects would have required (1) retrieving, analysing and reporting on different types of data (e.g. correlation data needed for comparison with other outcome measures, Mokkink et al., 2021) and (2) the inclusion of any longitudinal studies with at least two measurements (including intervention studies) reporting either the relationship between the change scores on the identified measures and other measures assessing similar constructs or the mean differences in change scores of the identified measures between different groups. Thus, including these two aspects in this review would have required several types of different search strategies, eligibility criteria and reporting. This would have made the review unmanageable in both length and complexity. Consequently, the findings on responsiveness of CM measures are limited to intervention studies only. Next, this review evaluated responsiveness based on only the mean differences between before and after interventions, which did not investigate the differences between baseline and follow-up. Although more than two measurement occasions can be a good strategy to avoid measurement error in detecting the true difference or change due to an intervention (Barkaoui, 2014), most of the included studies tended to measure CM at two time points (before and after interventions) without follow-up. Lastly, feasibility of measures and interpretability of change scores were also outside the scope of this review as neither feasibility nor interpretability are considered psychometric properties according to the COSMIN taxonomy, even though they are important characteristics to consider when selecting the most suitable measures (Mokkink, Prinsen, et al., 2018; Prinsen et al., 2018). One aspect of feasibility (i.e. cost of a measure), however, is described in Table 1.

### Implications for Future Research and Practice

From the findings on the methodological quality of the included studies in this systematic review, three implications for future research and practice arise. First, future studies on responsiveness to compare changes before and after parenting interventions using parent- or caregiver-reported CM measures are encouraged to calculate and report the effect sizes, in addition to *p*-values. This is also in line with the recommendations of *Reporting Standards for Research in Psychology* by the American Psychological Association (APA, 2008). Next, to estimate unbiased effect sizes on responsiveness, more RCT studies using parent- or caregiver-reported CM measures should be conducted; more than two measurement occasions (including follow-up) should also be considered a good strategy to avoid measurement error in detecting the true difference or change due to an intervention (Barkaoui, 2014). Third, to establish evidence-based parenting interventions for CM prevention, the selection of CM measures to be used in parenting interventions should not be based simply on their popularity, but on their psychometric evidence evaluating the responsiveness. The evaluation of responsiveness is recommended to be conducted using the COSMIN guidelines, which is a benchmark in the field of systematic review for evaluating measures’ psychometric quality due to its comprehensiveness and standardisation (Aromataris & Munn, 2020; Rosenkoetter & Tate, 2018). Lastly, for data on the responsiveness of a measure’s subscales to be meaningful, the internal structure of the measure should be confirmed using appropriate statistical analyses (i.e. factor analysis and internal consistency using Cronbach’s alpha per subscale) resulting in subscales measuring a single underlying construct. For five measures (APT, FM-CA, MCNS, POQ and PRCM) in particular, the internal structure is yet to be confirmed before further assessment of study quality and responsiveness is meaningful.

From the findings on the responsiveness of the included measures in this systematic review, another three implications for future research and practice arise. First, all overall scales or subscales of the 15 included measures need additional responsiveness studies due to lacking or low-quality evidence to support the quality of their responsiveness, with the exception of the Physical Abuse subscale of ICAST-Trial which demonstrated high-quality evidence. Next, because of high-quality evidence supporting its sufficient responsiveness, the Physical Abuse subscale of ICAST-Trial could be recommended for use in parenting interventions to reduce physical abuse to their children. Lastly, future research needs to perform subgroup analyses to investigate whether the characteristics of samples (e.g. level of income and gender) and intervention (e.g. dosage and timing) contribute to the substantial heterogeneity in effect sizes on responsiveness of parent- or caregiver-reported CM measures (e.g. AAPI-2, CTSPC, ICAST-Trial and PRCM reporting moderate to high heterogeneity in responsiveness across studies). The subgroup analyses may contribute to the selection and use of more culturally and contextually appropriate measures on CM in parenting interventions to reduce CM by parents.

This review used the WHO’s definition of CM focusing on four subtypes of CM perpetrated by parents or caregivers because it is the most commonly used definition internationally (Di et al., 2018; Sahagún-Morales et al., 2021) and most existing CM measures have been developed based on this definition; future review should consider using an expanded definition, including either CM perpetrated by neighbours and peers and children witnessing intimate partner violence (IPV) between parents (Finkelhor et al., 2005, 2011). Exposure to IPV may be considered a form of emotional abuse as it causes children psychological harm (e.g. fear and anxiety) when they witness hitting and yelling between parents. Furthermore, while most types of CM are perpetrated by parents or caregivers, sexual abuse is mainly perpetrated by peers or adults other than the child’s parents or caregivers (Brassard & Donovan, 2006; Somer & Braunstein, 1999). As multiple and distinct types of CM tend to occur simultaneously, CM measures based on the expanded definition of CM may help capture change in a child’s CM experience more sensitively than the measures based on single or limited types or CM by parents of caregivers (Finkelhor et al., 2005, 2011). Therefore, to capture change of a child’s CM experiences fully and sensitively, future studies on the responsiveness of CM measures should consider the expanded definition of CM.

## Conclusion

This systematic review evaluated the responsiveness of 15 parent- or caregiver-reported measures on CM using the COSMIN guidelines. Evidence concerning responsiveness was limited and mostly of lower quality. Based on current available evidence on responsiveness, only one subscale (Physical Abuse subscale of ICAST-Trial) of all included measures can be recommended as the most suitable measure of physical abuse in parenting interventions to reduce CM by parents. All other overall scales or subscales of the included measures were identified as promising but would still need further studies on their responsiveness before their use in clinical practice and research can be recommended.

## Supplemental Material

Supplemental Material - A Systematic Review on Evaluating Responsiveness of Parent- or Caregiver-Reported Child Maltreatment Measures for Interventions Click here for additional data file. Supplemental Material for A Systematic Review on Evaluating Responsiveness of Parent- or Caregiver-Reported Child Maltreatment Measures for Interventions by Sangwon Yoon, Renée Speyer, Reinie Cordier, Pirjo Aunio, and Airi Hakkarainen in Trauma, Violence, & Abuse
