# Supplementary material for: A Systematic Review on Evaluating Responsiveness of Parent- or Caregiver-Reported Child Maltreatment Measures for Interventions
Source: Trauma Violence Abuse. 2022 May 22;24(4):2297–318. doi: 10.1177/15248380221093690 (PMC10518736; doi:10.1177/15248380221093690)
Supplement: Supplemental Material - A Systematic Review on Evaluating Responsiveness of Parent- or Caregiver-Reported Child Maltreatment Measures for Interventions [file sj-pdf-1-tva-10.1177_15248380221093690.pdf]

Online Supplemental Materials

Table S1. *PRISMA Checklist.*

SECTION/TOPIC	#	CHECKLIST ITEM	REPORTED ON PAGE #
**TITLE**			
Title	1	Identify the report as a systematic review, meta-analysis, or both.	1
**ABSTRACT**			
Structured summary	2	Provide a structured summary including, as applicable: background; objectives; data sources; study eligibility criteria, participants, and interventions; study appraisal and synthesis methods; results; limitations; conclusions and implications of key findings; systematic review registration number.	1-2
**INTRODUCTION**			
Rationale	3	Describe the rationale for the review in the context of what is already known.	3-7
Objectives	4	Provide an explicit statement of questions being addressed with reference to participants, interventions, comparisons, outcomes, and study design (PICOS).	7-8
**METHODS**			
Protocol and registration	5	Indicate if a review protocol exists, if and where it can be accessed (e.g., Web address), and, if available, provide registration information including registration number.	N/A
Eligibility criteria	6	Specify study characteristics (e.g., PICOS, length of follow-up) and report characteristics (e.g., years considered, language, publication status) used as criteria for eligibility, giving rationale.	9
Information sources	7	Describe all information sources (e.g., databases with dates of coverage, contact with study authors to identify additional studies) in the search and date last searched.	9
Search	8	Present full electronic search strategy for at least one database, including any limits used, such that it could be repeated.	9
Study selection	9	State the process for selecting studies (i.e., screening, eligibility, included in systematic review, and, if applicable, included in the meta-analysis).	9-10
Data collection process	10	Describe method of data extraction from reports (e.g., piloted forms, independently, in duplicate) and any processes for obtaining and confirming data from investigators.	10
Data items	11	List and define all variables for which data were sought (e.g., PICOS, funding sources) and any assumptions and simplifications made.	10-15
Risk of bias in individual studies	12	Describe methods used for assessing risk of bias of individual studies (including specification of whether this was done at the study or outcome level), and how this information is to be used in any data synthesis.	10-12
Summary measures	13	State the principal summary measures (e.g., risk ratio, difference in means).	9
Synthesis of results	14	Describe the methods of handling data and combining results of studies, if done, including measures of consistency (e.g., I^2) for each meta-analysis.	13
Risk of bias across studies	15	Specify any assessment of risk of bias that may affect the cumulative evidence (e.g., publication bias, selective reporting within studies).	14
Additional analyses	16	Describe methods of additional analyses (e.g., sensitivity or subgroup analyses, meta-regression), if done, indicating which were pre-specified.	14

(Continued)

Table S1. *Continued.*

SECTION/TOPIC	#	CHECKLIST ITEM	REPORTED ON PAGE #
**ADDITIONAL ANALYSIS**			
Study selection	17	Give numbers of studies screened, assessed for eligibility, and included in the review, with reasons for exclusions at each stage, ideally with a flow diagram.	15-16
Study characteristics	18	For each study, present characteristics for which data were extracted (e.g., study size, PICOS, follow-up period) and provide the citations.	16
Risk of bias within studies	19	Present data on risk of bias of each study and, if available, any outcome level assessment (see item 12).	16
Results of individual studies	20	For all outcomes considered (benefits or harms), present, for each study: (a) simple summary data for each intervention group (b) effect estimates and confidence intervals, ideally with a forest plot.	16-17
Synthesis of results	21	Present results of each meta-analysis done, including confidence intervals and measures of consistency.	17-18
Risk of bias across studies	22	Present results of any assessment of risk of bias across studies (see Item 15).	17-18
**DISCUSSION**			
Summary of evidence	24	Summarize the main findings including the strength of evidence for each main outcome; consider their relevance to key groups (e.g., healthcare providers, users, and policy makers).	18-26
Limitations	25	Discuss limitations at study and outcome level (e.g., risk of bias), and at review-level (e.g., incomplete retrieval of identified research, reporting bias).	23-24
Conclusions	26	Provide a general interpretation of the results in the context of other evidence, and implications for future research.	26
**FUNDING**			
Funding	27	Describe sources of funding for the systematic review and other support (e.g., supply of data); role of funders for the systematic review.	N/A

Table S2. *Database Search Strategies.*

Database	Search Terms (Free text words)	Number of records
**CINAHL**	(((Adult Adolescent Parenting Inventory) OR (Adult-Adolescent Parenting Inventory)) AND Time limit 1999-Current) OR (Analog Parenting Task) OR (Child Neglect Questionnaire) OR (Child Neglect Scales) OR (Child Trauma Screen) OR ((Conflict Tactics Scales) AND (child and (parent or parents))) OR ((Family Maltreatment) AND Time limit 2019-Current) OR ((Child Abuse Screening) AND Time limit 2018-Current) OR (Intensity of Parental Punishment Scale) OR ((Mother-Child Neglect Scale) or (Mother Child Neglect Scale)) OR ((Parent-Child Aggression Acceptability Movie) OR (Parent Child Aggression Acceptability Movie)) OR (Parent Opinion Questionnaire) OR (Parental Response to Child Misbehavior) OR (Shaken Baby Syndrome awareness assessment))	195
**Embase**	*As per CINAHL*	116
**ERIC**	*As per CINAHL*	50
**PsycINFO**	*As per CINAHL*	1,031
**PubMed**[a]	*As per CINAHL*	129
**Sociological Abstracts**[a]	*As per CINAHL*	63

Notes. All searches performed on the 15th and 16th of January 2020 with an update on the 23rd of March 2021.

[a] Search terms in PubMed and Sociological Abstracts are same as in CINAHL except using double quotation marks before and after name of measures.

Table S3. *Risk of Bias checklist for assessing the methodological quality of studies adapted from the COSMIN manual for systematic reviews of measures (Mokkink et al., 2018).*

Psychometric property	Aspect	Standard[a]	Item description
Responsiveness	Comparison before and after an intervention	Design requirements	Was an adequate description provided of the intervention given?
		Statistical methods	Was the statistical method appropriate for the hypotheses to be tested?
		Other flaws	Were there any other important flaws in the design or statistical methods of the study?

Note. AUC = Area Under the Curve; The Risk of Bias checklist was used for assessing the methodological quality of studies (Step 2 in Figure 1).

[a] Each standard on methodological quality was rated using a four-point rating scale: inadequate, doubtful, adequate, and very good; The overall methodological quality per study was determined calculating a percentage of the ratings (Cordier et al., 2015): inadequate = 0–25%, doubtful = 25.1–50%, adequate = 50.1–75%, and very good = 75.1–100%.

Table S4. *Criteria for good responsiveness adapted from the COSMIN manual for systematic reviews of measures (Mokkink et al., 2018).*

Psychometric property	Aspect	Rating[a]	Quality criteria[b]
Responsiveness	Comparison before and after an intervention	+	Meaningful changes in scores before and after intervention (e.g., Hedges' *g* ≥0.50)
		?	Not all information for '+' reported (e.g., lack of information to calculate Hedges' *g*)
		-	Criteria for '+' not met (e.g., Hedges' *g* < 0.50)
		NR	No information found on responsiveness

Note. AUC = Area Under the Curve; The criteria for good responsiveness was used for rating the results of single studies on responsiveness (Step 3.1 of Figure 1) and rating the pooled results of all studies per measure (Step 3.2 of Figure1).

[a] + = Sufficient, - = Insufficient, ? = Indeterminate, and NR = Not Reported.

[b] The quality criterion for good responsiveness on comparison of change scores before and after intervention was determined as medium effect size (Hedges' *g* = 0.5) using (Cohen, 1988) conventions to interpret effect size, which was decided by the review team for this current review as suggested by the COSMIN manual (Mokkink et al., 2018).

Table S5. *Modified GRADE approach for grading the quality of evidence on responsiveness per measure adapted from the COSMIN manual for systematic reviews of measures (Mokkink et al., 2018).*

Level of evidence quality (sum of scores per factor)	Factor	Score	Criteria
**High (0)**	*Risk of bias*	0	Multiple studies of at least adequate methodological quality<br>*OR*<br>One study of very good methodological quality
**Moderate (-1)**		-1	Multiple studies of doubtful methodological quality<br>*OR*<br>Only one study of adequate methodological quality
**Low (-2)**		-2	Multiple studies of inadequate methodological quality<br>*OR*<br>Only one study of doubtful methodological quality
**Very low (< -3)**		-3	Only one study of inadequate methodological quality
	Inconsistency[a]	0	Low heterogeneity in results across studies (0% ≤ I^2 < 50%)
		-1	Moderate heterogeneity in results across studies (50% ≤ I^2 < 75%)
		-2	High heterogeneity in results across studies (75% ≤ I^2)
	Imprecision	0	Pooled sample sizes of all individual studies > 100
		-1	Pooled sample sizes of all individual studies = 50–100
		-2	Pooled sample sizes of all individual studies = n < 50
	Indirectness	0	All studies addressing construct or target population of the review
		-1	At least one study not addressing construct or target population of the review, but not all
		-2	All studies not addressing construct or target population of the review

Note. The modified GRADE approach was used for grading the quality of summarized evidence on responsiveness (Step 3.3 of Figure 1); The starting point of evidence quality is 'high' quality of evidence; the level of evidence quality is downgraded by the sum of scores per factor.

[a] The criterion for inconsistency was determined by the review team for this current review as suggested by the COSMIN manual (Mokkink et al., 2018), et al., 2018); The review team decided to evaluate inconsistency or heterogeneity in results across studies using *I-squared* (I^2) statistic that is the percentage of the total variability in a set of effect sizes across the studies due to heterogeneity; Values of less than 50%, 50% to 74%, and higher than 75% denote low, moderate, and high heterogeneity, respectively (Higgins et al., 2003).

Table S6. *Descriptions of included articles on responsiveness of measures for the assessment of child maltreatment.*

Measure	Study	Purpose of study	Name of Intervention	Sample allocation[a]	Sample size[b]	Study population	Percentage of Female	Age		
								Range	Mean	Standard Deviation
**AAPI-2**	Akai et al. (2008)	To evaluate the effectiveness of an intervention designed to improve early parenting	My Baby and Me	Random	23	Mothers at risk	100	15–38	22.81	5.07
	Alvarez et al. (2018)	To examine the components affecting the quality of the implementation and their impact on the outcomes of a parenting program	Growing Up Happily in the Family	Non-random	133	Parents with children aged 0 to 5 years	90.3	NR	32.85	8.36
	Axford et al. (2020)	To evaluate effectiveness of a therapeutic parenting program for parents of children with behavioural and emotional difficulties	Inspiring Futures	Random	134	Parents of children aged 6 to 11 years with behavioural and emotional difficulties	45.1	NR	NR	NR
	Barden et al. (2015)	To investigate the effectiveness of a relationship education on increasing positive parenting attitudes	Becoming Parents Program	Non-random	140	Economically strained couples with children	50	NR	NR	NR
	Barnes et al. (2017)	To determine the effectiveness and cost-effectiveness of a group-based parenting program in reducing risk factors for maltreatment	Group Family Nurse Partnership	Random	75	Mothers from pregnancy to the first year postpartum	100	NR	21	1.8
	Barnet et al. (2007)	To evaluate the impact of a community-based home-visiting program on poor parenting and other risk factors in pregnant adolescents	Home-Visiting Program	Random	31	Pregnant adolescents aged 12 to 18 years	100	NR	16.4	1.4
	Benzies et al. (2011)	To examine the effects of a two-generation, multi-cultural preschool program on children of Aboriginal heritage and their caregivers	One World	Non-random	23	Caregivers of aboriginal preschool children	NR	NR	30	5.76
	Benzies et al. (2014)	To evaluate a single-site, two-generation preschool demonstration program for low-income families in Canada	Nobody's Perfect; 1-2-3 Magic	Non-random	67	Low-income parents of preschool children	NR	8–46	30.82	6.3
	Berry et al. (2007)	To evaluate the effectiveness of a reunification program in increasing rates of reunification for foster children with their birth parents	Intensive Reunification Program	Non-random	4	Biological parents served a program for reunification with their children in foster care	NR	NR	NR	NR
	Burton et al. (2018)	To evaluate the impact of a parenting program for parents of children with developmental disabilities on nurturing parenting skills	Nurturing Program for Parents and Their Children with Special Needs and Health Challenges	Random	20	Parents of children with development disabilities	97.6	NR	NR	NR
	Clark et al. (2013)	To examine the effect of Love's Cradle relationship enhancement intervention on positive non-abusive parenting attitudes	Love's Cradle	Non-random	69	Low-income pregnant or postpartum (maximum of 3 months post-delivery) adult couples	50	NR	28.60	7.27
	Conn et al. (2018)	To assess the impacts of Incredible Years intervention on child behaviour, foster parent stress and attitudes, and perceived effect on parenting	Incredible Years	Random	16	Foster parents of children aged 2–7 years	81.3	NR	NR	NR

(Continued)

Table S6. *Continued.*

Measure	Study	Purpose of study	Name of Intervention	Sample allocation[a]	Sample size[b]	Study population	Percentage of Female	Age		
								Range	Mean	Standard Deviation
**AAPI-2**	Conners et al. (2006)	To examine the impact of a comprehensive, residential substance abuse treatment program for pregnant and parenting women on substance use, employment, legal involvement, mental health symptoms, risky sexual behaviour, and parenting attitudes	Residential treatment for substance abuse problems	Non-random	200	Pregnant women and mothers with substance abuse	100	NR	29.8	7.2
	Cullen et al. (2010)	To examine the effects of a home visitation program on the parenting attitudes and practices of at-risk parents	Healthy Families America home visitation program	Non-random	55	At-risk parents	100	NR	NR	NR
	Estefan et al. (2013)	To explore the family stressors in parents involved in the child welfare system who have been referred to an intensive therapeutic parenting program, and the relationship of those stressors to change in parenting attitudes	Nurturing Parenting Program	Non-random	94	Parents involved in the child welfare system	52.1	NR	NR	NR
	Farber (2009)	To assess the effects of parent mentoring and guidance programs on changes in parenting and child outcomes	Well-Baby Care; Brazelton Touchpoints Training	Non-random	30	Low-income Latino and African American mothers	100	NR	23	5.6
	Galanter et al. (2012)	To evaluate the effect of a parent–child interaction therapy delivered in-home by community agency therapists on changes in parenting behaviour and attitudes of parents	In-Home Parent–Child Interaction Therapy	Non-random	48	Parents at risk for child maltreatmen	88.0	NR	NR	NR
	Gibbs et al. (2008)	To evaluate the impact of a health camp psychosocial intervention on children with behavioural and emotional problems and the impact of a parenting programme of their parents.	Health camp intervention	Non-random	100	Parents of children with emotional and behaviour problems	89.0	NR	34.6	6.4
	Lavi et al. (2015)	To examine the potential impact of an evidence-based treatment for traumatized mother–child dyads on maternal functioning 6 months post-partum	Child-Parent Psychotherapy	Non-random	64	Pregnant women at risk for intimate partner violence	100	NR	27.5	8.9
	Lawson et al. (2012)	To examine the extent to which participation in a county-wide prevention program leads to improvements in protective factors associated with child abuse prevention	safe families	Non-random	1184	Mothers living in economically and socially vulnerable communities in urban region	100	NR	NR	NR

(Continued)

Table S6. *Continued.*

Measure	Study	Purpose of study	Name of Intervention	Sample allocation[a]	Sample size[b]	Study population	Percentage of Female	Age		
								Range	Mean	Standard Deviation
**AAPI-2**	LeCroy and Judy (2011)	To examine the effectiveness of home visiting on improving parental, child, and maternal outcomes and preventing child abuse and neglect	Healthy Families Program	Random	92	Mothers at risk	100	NR	23.5	NR
	Maher et al. (2011)	To examine the relationship between program dosage and subsequent child maltreatment.	Nurturing Parenting Program	Random	442	Parents of young children (from infant to pre-school) referred to child welfare services	74	12–60	27.38	7.35
	Marcynyszyn et al. (2011)	To exam the effectiveness of an evidence-based parent education program in the context of a child welfare population, as well as implementation challenges and recommendations.	Incredible Years	Non-random	24	Caregivers involved in child welfare agencies	71	NR	36	NR
	McKelvey et al. (2012)	To examine the impact of a home visiting intervention on adolescent mothers’ parenting attitudes	Healthy Families America intervention	Non-random	93	Low income Adolescent mothers	100	NR	17.3	1.4
	Miller et al. (2014)	To assess mothers' needs and interests regarding parenting while they were incarcerated, adapt the program to address those needs, and establish intervention delivery and evaluation methods in collaboration with a community-based agency	Parenting While Incarcerated	Non-random	22	Mothers of children under 18 at the local county jail	100	21–48	31	6.8
	Palusci et al. (2008)	to measure the effects of a formal parenting education program offered to several high-risk groups, including incarcerated and residential substance abuse treatment populations, before maltreatment occurred	Helping Your Child Succeed based on Family Nurturing Program	Non-random	781	Parents enrolled in diverse rehabilitation services to reduce substance abuse, violence, and mental health problems in county jail and community	44	NR	33.2	NR
	Renzaho and Sonia (2011)	To evaluate the impact of a culturally appropriate parenting program to reduce intergenerational conflicts and enhance family cohesion and wellbeing among sub-Saharan African refugees and migrants living in Australia.	African Migrant Parenting Program	Non-random	39	African migrant and refugee parents in Australia	54	19–55	33.4	10.9
	Robbers (2008)	To evaluate the effect of a multifaceted intervention program operating on improving parenting skills of teenage mothers and their male partners	Caring Equation	Non-random	194	Adolescent parents	73	14–20	16.72	2.18
	Rodriguez et al. (2010)	To examine the effectiveness of a home visiting program in promoting parenting competencies and preventing maladaptive parenting behaviours in mothers at risk for child abuse and neglect	Healthy Families New York	Random	255	Mothers at risk for child abuse and neglect	100	NR	22.5	5.8

(Continued)

Table S6. *Continued.*

Measure	Study	Purpose of study	Name of Intervention	Sample allocation[a]	Sample size[b]	Study population	Percentage of Female	Age		
								Range	Mean	Standard Deviation
**AAPI-2**	Sangalang and Kathleen (2005)	To examine the effect of parenting case management program on substance use, contraceptive behaviour, and parenting knowledge	North Carolina's Adolescent Parenting Program	Non-random	91	Pregnant and parenting adolescents	100	12–18	15.9	1.2
	Sawasdipanich et al. (2010)	To examine the effects of a cognitive adjustment program on parental attitudes toward child rearing and the potential for this abuse	Full Love in the Family Protects Your Kids	Random	53	Thai parents of children aged 1 to 6 years	79	NR	NR	NR
	Schilling et al. (2017)	To measure impact of group parent training designed to teach positive parenting skills on child behaviour and parenting attitudes	Child–Adult Relationship Enhancement in Primary Care (PriCARE)	Random	80	Parents of children 2 to 6 years old with behaviour difficulties	95	NR	NR	NR
	Scudder et al. (2014)	To explore the effectiveness of two facility-based group parenting models in enhancing parent-reported and observed parenting outcomes	Parent–Child Interaction Therapy	Random	39	Mothers (of a child aged 2 to 12) incarcerated at a state correctional facility	100	NR	31.31	4.69
	Strickler et al. (2018)	To compare the effect of an enhanced pre-service training developed for treatment parents on their parenting attitudes, personal dedication and willingness to provide foster care, and licensing rates	Pressley Ridge's Treatment Foster Care	Non-random	66	Prospective treatment foster parents	63	NR	48.34	13.00
	Stover et al. (2019)	To evaluate a residential substance misuse treatment program for fathers, integrated treatment for intimate partner violence and child maltreatment	Fathers for Change (F4C)	Random	34	Fathers registered in residential substance use treatment programs	0	23-62	36.82	9.07
	Suess et al. (2016)	To examine the effect of an attachment-based early intervention program on attachment security, parental stress, attitudes, and depression of German mothers	Steps Toward Effective and Enjoyable Parenting	Non-random	54	Young high-risk mothers	100	NR	18.08	NR
	Thomas and Stephen (2004)	To examine the effectiveness of a comprehensive psychoeducational intervention on depression, self-esteem, and parenting attitudes/beliefs of at-risk pregnant and parenting adolescents	Residential Treatment Facility	Non-random	5	Pregnant and parenting Adolescents	100	14-20	16.8	1.3
	Twomey et al. (2010)	To exam the impact of maternal participation in a multidisciplinary therapeutic approach for perinatal substance users on maternal functioning, infant developmental, and permanency outcomes	Family Treatment Drug Court	Non-random	52	Perinatal substance user mothers participating in family treatment drug court	100	19–45	29.2	6.3

(Continued)

Table S6. *Continued.*

Measure	Study	Purpose of study	Name of Intervention	Sample allocation[a]	Sample size[b]	Study population	Percentage of Female	Age		
								Range	Mean	Standard Deviation
**AAPI-2**	Waters et al. (2015)	To investigate maternal sensitivity in a treatment-seeking sample of predominately Latina, low-income pregnant women with histories of interpersonal trauma exposure	Perinatal Child–Parent Psychotherapy (P-CPP)	Non-random	51	Latina, low-income pregnant women	100	18–40	27.08	5.66
	Waterston et al. (2009)	To evaluate the effect of a parenting newsletter, sent monthly to the parents' home from birth to 1 year, on maternal well-being and parenting style	Baby Express	Random	81	First-time mothers	100	NR	29.4	5.8
	Wood et al. (2020)	To measure the impact of a primary care program on disruptive child behaviours, parenting stress, and parenting attitudes	Primary Care (PriCARE)	Random	105	Caregivers of children ages 2 to 6 years with behaviour difficulties	96	NR	NR	NR
	Zajicek-Farber (2010)	To evaluate the impact of an individualized parent mentoring on parenting practices and knowledge of age-appropriate nurturing and emotionally sensitive caregiving	Parent Mentoring Intervention	Non-random	35	Pregnant mothers with high-risk in urban settings	100	NR	23	5.6
	Zolnoski et al. (2012)	To assess the effect of a mixed home visitation parenting program on addressing family need and the risk for child maltreatment	Healthy Families America; Parents as Teachers	Non-random	13	Parents registered in home visiting program	82	21–62	32.5	11.1
**APT**	Holland and Holden (2016)	To evaluate the efficacy of a motivational interviewing approach in changing positive attitudes toward corporal punishment behavioural intentions, and behaviour	Motivational Interviewing	Random	21	Mothers of children ages 3 to 5	100	22–44	32.37	6.3
**CNQ**	No study included	NR	NR	NR	NR	NR	NR	NR	NR	NR
**CNS-MMS**	No study included	NR	NR	NR	NR	NR	NR	NR	NR	NR
**CTS-ES**	No study included	NR	NR	NR	NR	NR	NR	NR	NR	NR
**CTSPC**	Dobowitz et al. (2012)	To examine the effectiveness of a paediatric primary care program on reducing child maltreatment	Safe Environment for Every Kid	Random	583	Mothers of children ages 0 to 5 years	100	NR	33.4	5.7
	Feinberg et al. (2016)	To test the short-term efficacy of a brief, universal, transition-to-parenthood intervention	Family Foundations	Random	169	Couples expecting their first child	50	NR	30.10	4.93
	Fowler and Michael (2017)	To test whether permanent housing plus housing case management reduces child maltreatment among families at risk of out-of-home placement compared to housing case management alone	Family Unification Program; Housing Advocacy Program	Random	68	Homeless and child welfare-involved parents	92	NR	32.0	8.5

(Continued)

Table S6. *Continued.*

Measure	Study	Purpose of study	Name of Intervention	Sample allocation[a]	Sample size[b]	Study population	Percentage of Female	Age		
								Range	Mean	Standard Deviation
**CTSPC**	Guterman et al. (2013)	To examine the benefits of home-based paraprofessional parent aide services in reducing physical abuse and neglect risk in high-risk parents	Parent Aide; Case Management	Random	73	High-risk parents	100	NR	29.2	0.9
	Guterman et al. (2018)	To assess the feasibility, acceptability, and preliminary outcomes of an evidence-based perinatal home visitation program	Dads Matter	Non-random	23	Biological parents in vulnerable families	50	NR	22.50	6.29
	Knox and Burkhart (2014)	To examine the factors related to attrition and treatment outcomes in a family violence and child abuse prevention program for parents and caregivers of young children.	ACT-Raising Safe Kids	Non-random	60	Parents and caregivers of young children	75	NR	36.41	8.93
	Lindhiem et al. (2014)	To compare changes in two different assessments (the absolute frequency method and the relative frequency method for quantifying parenting practices) in response to treatment	Parent-Management Training	Non-random	139	Parents of children with disruptive behaviour problems	NR	NR	NR	NR
	McDonell et al. (2015)	To evaluate the effect of a multi-year comprehensive community-based initiative on preventing child maltreatment and improve children's safety.	Strong Communities for Children	Random	229	Parents or caregivers of a child aged 10 or younger	72.5	NR	35.9	8.7
	Ondersma et al. (2017)	To test the effectiveness of a multicomponent computer-based parenting program to prevent child maltreatment	e-Parenting Program; Early home visitation	Random	112	At-risk mothers	100	NR	23.8	4.8
	Oveisi et al. (2010)	To assess whether primary health care settings can be used to engage and provide a preventive intervention to mothers of young children	SOS (helps for parents) Program	Random	108	Iranian mothers of young children (age 2 to 6)	100	NR	29.8	4.49
	Portnoy et al. (2018)	To evaluating the effect of omega-3 supplementation to reducing intimate partner violence and child maltreatment among adult caregivers	Omega-3	Random	94	Caregivers of young children in Maritius	89	NR	38.21	2.99
	Self-brown et al. (2017)	To examine the acceptability and initial efficacy of an augmented version of the evidence-based child maltreatment prevention program, SafeCare, for improving father parenting skills and reducing maltreatment risk	SafeCare® Dad to Kids	Random	50	At-risk fathers	0	NR	30.05	7.75

(Continued)

Table S6. *Continued.*

Measure	Study	Purpose of study	Name of Intervention	Sample allocation[a]	Sample size[b]	Study population	Percentage of Female	Age		
								Range	Mean	Standard Deviation
**CTSPC**	Shaffer et al. (2013)	To examine pre–post treatment changes for a modular intervention that has previously demonstrated significant clinical improvements in child behaviour and maintenance of these effects	Modular Intervention	Random	137	Parents of children ages 6 to11 with disruptive behaviour problems	15	NR	NR	NR
	Swenson et al. (2010)	To evaluate effectiveness of Multisystemic Therapy for Child Abuse and Neglect for physically abused youth and their families	Multisystemic Therapy for Child Abuse and Neglect	Random	43	Parents of physically abused youth	65.9	NR	40.82	11.15
	Wieling et al. (2015)	To assess the feasibility of providing a parenting intervention for war-affected families in Uganda	Enhancing Family Connection	Non-random	14	War-affected mothers in Northern Uganda	100	23–48	33.5	7.0
	Zoysa et al. (2015)	To exam the impact of an awareness raising program to reduce parental use of aversive disciplinary practices	Awareness Raising Program	Non-random	157	Sri Lankan parents	87.6	20–70	39.8	8.86
**FM-CA**	Slep et al. (2020)	To evaluate the effectiveness of a community-based framework to reduce adult substance misuse, intimate partner violence, child abuse, suicidality, and cumulative risk	NORTH STAR	Random	11,377	Military parents with children at US Air Force base	42	NR	32.61	7.65
**ICAST-Trial**	Meinick et al. (2018)	To evaluate the adaptation and the psychometric properties of the ISPCAN child abuse screening tool for use in trials (ICAST-Trial) among South African adolescents and their primary caregivers	Parenting for Lifelong Health	Random	240	Primary caregivers of South African adolescents	94.7	NR	49.4	14.69
	Shenderovich et al. (2019)	To examine whether the implementation measures in this study predict participant outcomes on child maltreatment and parenting behaviour	Sinovuyo Teen	Random	270	Caregivers of South African adolescents aged 10-18	97	NR	49	15.2
	Cluver et al. (2018)	To assess the impact of a parenting programme for adolescents in low-income and middle-income countries, on abuse and parenting practices	Sinovuyo Teen	Random	270	Caregivers reporting conflict with their adolescent children (aged 10–18)	97	NR	48.79	
	Lachman et al. (2020)	To evaluate the effectiveness of an intervention combining parenting and economic strengthening programmes to reduce violence against children for caregivers in rural Tanzania	Skillful Parenting & Agribusiness	Random	248	Parents with children aged 0–18 years in farming communities in Tanzania	55	NR	41.65	
**IPPS**	No study included	NR	NR	NR	NR	NR	NR	NR	NR	NR

(Continued)

Table S6. *Continued.*

Measure	Study	Purpose of study	Name of Intervention	Sample allocation[a]	Sample size[b]	Study population	Percentage of Female	Age		
								Range	Mean	Standard Deviation
**MCNS**	Gallitto et al. (2020)	To investigate the impact of the SafeCare program on parenting behaviours in child welfare-involved families	SafeCare®	Non-random	68	Child welfare-involved caregivers from Ontario	82.9	NR	28.1	8.9
**MCNS-SF**	No study included	NR	NR	NR	NR	NR	NR	NR	NR	NR
**P-CAAM**	No study included	NR	NR	NR	NR	NR	NR	NR	NR	NR
**POQ**	Sanders et al. (2004)	To exam whether parental attributional retraining and anger management enhance the effects of the Triple P-Positive Parenting Program with parents at risk of child maltreatment	Triple P-Positive Parenting Program; Enhanced group Behavioral Family Intervention	Non-random	35	Parents at risk for child maltreatment	NR	NR	33.33	5.37
	Vorhies et al. (2009)	To evaluate the effectiveness of a residential program with comprehensive wrap-around services for pregnant and parenting foster care youth with severe mental illness or severe emotional disturbance who are preparing to transition to independent living	Thresholds Mothers' Project	Non-random	17	Pregnant and parenting foster care female youth with severe mental illness	100	NR	19.31	1.23
**PRCM**	Holland et al. (2016)	To evaluate the efficacy of a motivational interviewing approach in changing positive attitudes toward corporal punishment behavioural intentions, and behaviour	Motivational Interviewing	Random	21	Mothers of children ages 3 to 5	100	22–44	32.37	6.3
	Caughy et al. (2003)	To exam the effects of Healthy Steps on discipline strategies of parents of young children	Healthy Steps	Random	134	Parents of children aged 16 to 37 months	NR	NR	NR	NR
**SBS-SF**	No study included	NR	NR	NR	NR	NR	NR	NR	NR	NR

Note. AAPI-2 = Adult Adolescent Parenting Inventory-2, APT = Analog Parenting Task, CNQ = Child Neglect Questionnaire, CNS-MMS = Child Neglect Scales-Maternal Monitoring and Supervision Scale, CTS-ES = Child Trauma Screen-Exposure Score, CTSPC = Conflict Tactics Scales: Parent-Child version, FM-CA = Family Maltreatment-Child Abuse criteria, ICAST-Trial = ISPCAN (International Society for the Prevention of Child Abuse and Neglect) Child Abuse Screening Tool for use in Trials, IPPS = Intensity of Parental Punishment Scale, MCNS = Mother-Child Neglect Scale, MCNS-SF = Mother-Child Neglect Scale-Short Form, P-CAAM = Parent-Child Aggression Acceptability Movie task, POQ = Parent Opinion Questionnaire, PRCM = Parental Response to Child Misbehavior questionnaire, SBS-SV = Shaken Baby Syndrome awareness assessment-Short Version; NR = Not Reported.

[a] Random sample allocation indicates that the sample is randomly allocated to an intervention or control group; Non-random sample allocation indicates that the sample is not randomly allocated to an intervention or control group (Altman, 1991).

[b] Sample size is the total number of sample completing the measures both before and after intervention in treatment group.

Table S7. *Single analysis at scale level results and ratings on responsiveness: Detailed findings for Step 3.1 in Figure 1.*

Measure: Overall scale / subscale[a]	Reference	Methodological quality of study[b]	Statistical method of study[c]	Sample allocation[d]	Sample size	Study population	Result of each study Hedges' *g* effect size[e] (95% CI)	Rating[f] on result per study
**AAPI-2: Overall scale**	Akai et al. (2008)	Adequate	*P*-value	Random	23	Mothers	**1.501** (0.417 – 2.584)	+
	Alvarez et al. (2018)	Very good	Effect size	Non-random	133	Parents	**0.303** (0.121 – 0.485)	-
	Axford et al. (2020)	Very good	*P*-value	Random	134	Parents	**-0.205** (-0.375 – -0.034)	-
	Barden et al. (2015)	Doubtful	Effect size	Non-random	140	Couples with children	**0.116** (-0.05 – 0.281)	-
	Barneset al. (2017)	Very good	Effect size	Random	75	Mothers	**0.36** (0.058 – 0.663)	-
	Barnetet al. (2007)	Adequate	*P*-value	Random	31	Pregnant adolescents	**0.492** (0.03 – 0.954)	-
	Benzies et al. (2011)	Doubtful	Effect size	Non-random	23	Caregivers	**0.111** (-0.259 – 0.481)	-
	Benzies et al. (2014)	Very good	*P*-value	Non-random	67	Parents	**0.136** (-0.103 – 0.375)	-
	Berry et al. (2007)	Doubtful	Effect size	Non-random	4	Parents	**NR**	?
	Burton et al. (2018)	Very good	Effect size	Random	20	Parents	**0.226** (-0.219 – 0.671)	-
	Clark et al. (2013)	Doubtful	*P*-value	Non-random	69	Couples with babies	**1.024** (0.718 – 1.329)	+
	Conn et al. (2018)	Doubtful	*P*-value	Random	16	Foster parents	**0.005** (-0.497 – 0.507)	-
	Conners et al. (2006)	Doubtful	*P*-value	Non-random	200	Mothers	**0.187** (0.048 – 0.326)	-
	Cullen et al. (2010)	Doubtful	*P*-value	Non-random	55	Mothers	**1.804** (1.378 – 2.23)	+
	Estefan et al. (2013)	Doubtful	Effect size	Non-random	94	Parents	**1.005** (0.829 – 1.18)	+
	Farber (2009)	Very good	Effect size	Non-random	30	Mothers	**0.774** (0.31 – 1.238)	+
	Galanter et al. (2012)	Adequate	*P*-value	Non-random	48	Parents	**0.476** (0.18 – 0.772)	-
	Gibbs et al. (2008)	Inadequate	Effect size	Non-random	100	Parents	**-0.001** (-0.2 – 0.199)	-
	Lavi et al. (2015)	Very good	*P*-value	Non-random	64	Pregnant women	**0.91** (0.607 – 1.213)	+
	Lawson et al. (2012)	Adequate	*P*-value	Non-random	1184	Mothers	**0.383** (0.324 – 0.442)	-
	LeCroy and Judy (2011)	Doubtful	Effect size	Random	92	Mothers	**-0.35** (-0.672 – -0.027)	-
	Maher et al. (2011)	Very good	*P*-value	Random	442	Parents	**-0.005** (-0.098 – 0.088)	-
	Marcynyszyn et al. (2011)	Doubtful	*P*-value	Non-random	24	Caregivers	**0.275** (-0.13 – 0.679)	-
	McKelvey et al. (2012)	Inadequate	*P*-value	Non-random	93	Adolescent mothers	**0.124** (-0.131 – 0.379)	-
	Miller et al. (2014)	Doubtful	*P*-value	Non-random	22	Mother	**0.162** (-0.243 – 0.568)	-
	Palusci et al. (2008)	Doubtful	*P*-value	Non-random	773	Parents	**NR**	?
	Renzaho and Sonia (2011)	Doubtful	*P*-value	Non-random	39	Parents	**0.732** (0.388 – 1.077)	+
	Robbers (2008)	Doubtful	*P*-value	Non-random	194	Adolescent parents	**0.655** (0.529 – 0.781)	+
	Rodriguez et al. (2010)	Adequate	Effect size	Random	255	Mothers	**0.049** (-0.098 – 0.196)	-
	Sangalang and Kathleen (2005)	Doubtful	*P*-value	Non-random	91	Adolescent parents	**0.297** (0.09 – 0.504)	-
	Sawasdipanich et al. (2010)	Very good	Effect size	Random	53	Parents	**0.539** (0.254 – 0.823)	+
	Schilling et al. (2017)	Adequate	*P*-value	Random	80	Parents	**0.37** (0.144 – 0.596)	-

(Continued)

Table S7. *(Continued).*

Measure: Overall scale / subscale[a]	Reference	Methodological quality of study[b]	Statistical method of study[c]	Sample allocation[d]	Sample size	Study population	Result of each study Hedges' *g* effect size[e] (95% CI)	Rating[f] on result per study
**AAPI-2: Overall scale**	Scudder et al. (2014)	Very good	Effect size	Random	39	Mothers	**0.463** (0.022 – 0.904)	-
	Stover et al. (2019)	Adequate	*P*-value	Non-random	34	Fathers	**0.446** (0.101 – 0.791)	-
	Strickler et al. (2018)	Very good	*P*-value	Random	66	Foster parents	**0.332** (0.084 – 0.579)	-
	Suess et al. (2016)	Inadequate	*P*-value	Non-random	54	Young mothers	**0.42** (0.067 – 0.774)	-
	Thomas and Stephen (2004)	Inadequate	Effect size	Non-random	5	Adolescent parents	**1.135** (0.142 – 2.128)	+
	Twomey et al. (2010)	Doubtful	Effect size	Non-random	52	Mothers	**-0.092** (-0.376 – 0.193)	-
	Waters et al. (2015)	Adequate	*P*-value	Non-random	51	Pregnant women	**0.895** (0.574 – 1.217)	+
	Waterston et al. (2009)	Very good	Effect size	Random	81	First-time mothers	**0.213** (-0.006 – 0.433)	-
	Wood et al. (2020)	Adequate	*P*-value	Random	105	Caregivers	**0.222** (0.028 – 0.415)	-
	Zajicek-Farber (2010)	Very good	Effect size	Non-random	35	Pregnant mothers	**1.411** (1.087 – 1.734)	+
	Zolnoski et al. (2012)	Adequate	*P*-value	Non-random	13	Parents	**0.037** (-0.479 – 0.553)	-
**AAPI-2: Inappropriate Expectations subscale**	Alvarez et al. (2018)	Very good	Effect size	Non-random	133	Parents	**0.14** (-0.03 – 0.31)	-
	Benzies et al. (2011)	Doubtful	*P*-value	Non-random	23	Caregivers	**0.217** (-0.082 – 0.516)	-
	Benzies et al. (2014)	Very good	Effect size	Non-random	67	Parents	**0.157** (-0.082 – 0.395)	-
	Burton et al. (2018)	Very good	Effect size	Random	20	Parents	**0.384** (-0.053 – 0.821)	-
	Clark et al. (2013)	Doubtful	Effect size	Non-random	69	Couples with babies	**0.126** (-0.108 – 0.36)	-
	Conn et al. (2018)	Doubtful	*P*-value	Random	16	Foster parents	**0.074** (-0.392 – 0.54)	-
	Conners et al. (2006)	Doubtful	*P*-value	Non-random	200	Mothers	**0.284** (0.144 – 0.425)	-
	Cullen et al. (2010)	Doubtful	*P*-value	Non-random	55	Mothers	**1.203** (0.859 – 1.547)	+
	Estefan et al. (2013)	Doubtful	*P*-value	Non-random	94	Parents	**1.145** (0.962 – 1.328)	+
	Galanter et al. (2012)	Adequate	Effect size	Non-random	48	Parents	**0.518** (0.221 – 0.815)	+
	Gibbs et al. (2008)	Inadequate	*P*-value	Non-random	100	Parents	**-0.392** (-0.594 – -0.19)	-
	LeCroy and Judy (2011)	Doubtful	*P*-value	Random	92	Mothers	**0.415** (0.129 – 0.702)	-
	Maher et al. (2011)	Very good	Effect size	Random	442	Parents	**-0.004** (-0.097 – 0.089)	-
	Marcynyszyn et al. (2011)	Doubtful	*P*-value	Non-random	24	Caregivers	**0.282** (-0.113 – 0.677)	-
	McKelvey et al. (2012)	Inadequate	*P*-value	Non-random	93	Adolescent mothers	**0.152** (-0.103 – 0.407)	-
	Miller et al. (2014)	Doubtful	*P*-value	Non-random	22	Mother	**0.405** (-0.015 – 0.825)	-
	Renzaho and Sonia (2011)	Doubtful	*P*-value	Non-random	39	Parents	**0.846** (0.491 – 1.201)	+
	Robbers (2008)	Doubtful	*P*-value	Non-random	194	Adolescent parents	**0.826** (0.711 – 0.941)	+
	Rodriguez et al. (2010)	Adequate	*P*-value	Random	255	Mothers	**0** (-0.147 – 0.147)	-
	Sangalang and Kathleen (2005)	Doubtful	*P*-value	Non-random	91	Adolescent parents	**0.26** (0.054 – 0.466)	-
	Schilling et al. (2017)	Adequate	Effect size	Random	80	Parents	**0.282** (0.061 – 0.504)	-

(Continued)

Table S7. *(Continued).*

Measure: Overall scale / subscale[a]	Reference	Methodological quality of study[b]	Statistical method of study[c]	Sample allocation[d]	Sample size	Study population	Result of each study Hedges' *g* effect size[e] (95% CI)	Rating[f] on result per study
**AAPI-2: Inappropriate Expectations subscale**	Scudder et al. (2014)	Very good	Effect size	Random	39	Mothers	**0.086** (-0.346 – 0.518)	-
	Strickler et al. (2018)	Very good	*P*-value	Non-random	66	Foster parents	**0.449** (0.199 – 0.7)	-
	Twomey et al. (2010)	Doubtful	Effect size	Non-random	52	Mothers	**-0.445** (-0.726 – -0.164)	-
	Waters et al. (2015)	Adequate	Effect size	Non-random	51	Pregnant women	**0.339** (0.117 – 0.561)	-
	Wood et al. (2020)	Adequate	*P*-value	Random	105	Caregivers	**0.211** (0.019 – 0.403)	-
	Zolnoski et al. (2012)	Adequate	*P*-value	Non-random	13	Parents	**0** (-0.509 – 0.509)	-
**AAPI-2: Lack of Empathy subscale**	Akai et al. (2008)	Adequate	*P*-value	Random	23	Mothers	**0.971** (0.025 – 1.917)	+
	Alvarez et al. (2018)	Very good	Effect size	Non-random	133	Parents	**1.204** (0.982 – 1.427)	+
	Axford et al. (2020)	Very good	*P*-value	Random	134	Parents	**-0.205** (-0.375 – -0.034)	-
	Barden et al. (2015)	Doubtful	*P*-value	Non-random	140	Couples with children	**0.042** (-0.122 – 0.207)	-
	Benzies et al. (2011)	Doubtful	Effect size	Non-random	23	Caregivers	**-0.079** (-0.437 – 0.278) 3735	-
	Benzies et al. (2014)	Very good	Effect size	Non-random	67	Parents	**0.971** (0.025 – 1.917)	+
	Burton et al. (2018)	Very good	Effect size	Random	20	Parents	**1.205** (0.982 – 1.427)	+
	Clark et al. (2013)	Doubtful	*P*-value	Non-random	69	Couples with babies	**0.043** (-0.122 – 0.207)	-
	Conn et al. (2018)	Doubtful	*P*-value	Random	16	Foster parents	**-0.08** (-0.437 – 0.278)	-
	Conners et al. (2006)	Doubtful	*P*-value	Non-random	200	Mothers	**0.127** (-0.111 – 0.364)	-
	Cullen et al. (2010)	Doubtful	*P*-value	Non-random	55	Mothers	**0.806** (0.317 – 1.296)	+
	Estefan et al. (2013)	Doubtful	Effect size	Non-random	94	Parents	**1.619** (1.262 – 1.976)	+
	Galanter et al. (2012)	Adequate	*P*-value	Non-random	48	Parents	**0.712** (0.185 – 1.238)	+
	Gibbs et al. (2008)	Inadequate	*P*-value	Non-random	100	Parents	**-0.061** (-0.199 – 0.077)	-
	LeCroy and Judy (2011)	Doubtful	Effect size	Random	92	Mothers	**1.492** (1.111 – 1.874)	+
	Maher et al. (2011)	Very good	*P*-value	Random	442	Parents	**0.945** (0.774 – 1.115)	+
	Marcynyszyn et al. (2011)	Doubtful	*P*-value	Non-random	24	Caregivers	**0.171** (-0.109 – 0.452)	-
	McKelvey et al. (2012)	Inadequate	*P*-value	Non-random	93	Adolescent mothers	**0.063** (-0.132 – 0.257)	-
	Miller et al. (2014)	Doubtful	*P*-value	Non-random	22	Mother	**0.343** (0.057 – 0.628)	-
	Renzaho and Sonia (2011)	Doubtful	*P*-value	Non-random	39	Parents	**0.006** (-0.087 – 0.099)	-
	Robbers (2008)	Doubtful	*P*-value	Non-random	194	Adolescent parents	**0.843** (0.388 – 1.297)	+
	Rodriguez et al. (2010)	Adequate	*P*-value	Random	255	Mothers	**0.043** (-0.103 – 0.190)	-
	Sangalang and Kathleen (2005)	Doubtful	*P*-value	Non-random	91	Adolescent parents	**0.506** (0.248 – 0.764)	+
	Schilling et al. (2017)	Adequate	Effect size	Random	80	Parents	**-0.39** (-0.809 – 0.029)	-
	Scudder et al. (2014)	Very good	Effect size	Random	39	Mothers	**0.749** (0.401 – 1.097)	+
	Strickler et al. (2018)	Very good	Effect size	Non-random	66	Foster parents	**0.543** (0.287 – 0.799)	+

(Continued)

Table S7. *(Continued).*

Measure: Overall scale / subscale[a]	Reference	Methodological quality of study[b]	Statistical method of study[c]	Sample allocation[d]	Sample size	Study population	Result of each study Hedges' *g* effect size[e] (95% CI)	Rating[f] on result per study
**AAPI-2: Lack of Empathy subscale**	Waterston et al. (2009)	Very good	Effect size	Random	81	First-time mothers	**-0.548** (-0.654 – -0.441)	-
	Wood et al. (2020)	Adequate	Effect size	Random	105	Caregivers	**0.145** (-0.046 – 0.336)	-
	Zolnoski et al. (2012)	Adequate	*P*-value	Non-random	13	Parents	**0.043** (-0.104 – 0.191)	-
**AAPI-2: Oppressing Children's Power and Independence subscale**	Alvarez et al. (2018)	Very good	Effect size	Non-random	133	Parents	**-0.205** (-0.375 – -0.034)	-
	Benzies et al. (2011)	Doubtful	*P*-value	Non-random	23	Caregivers	**0.269** (-0.117 – 0.654)	-
	Benzies et al. (2014)	Very good	Effect size	Non-random	67	Parents	**-0.087** (-0.324 – 0.151)	-
	Burton et al. (2018)	Very good	Effect size	Random	20	Parents	**-0.297** (-0.727 – 0.134)	-
	Clark et al. (2013)	Doubtful	Effect size	Non-random	69	Couples with babies	**0.546** (0.296 – 0.797)	+
	Conn et al. (2018)	Doubtful	*P*-value	Random	16	Foster parents	**0.027** (-0.438 – 0.492)	-
	Conners et al. (2006)	Doubtful	*P*-value	Non-random	200	Mothers	**0** (-0.138 – 0.138)	-
	Cullen et al. (2010)	Doubtful	*P*-value	Non-random	55	Mothers	**0.948** (0.633 – 1.264)	+
	Estefan et al. (2013)	Doubtful	*P*-value	Non-random	94	Parents	**0.831** (0.668 – 0.995)	+
	Gibbs et al. (2008)	Inadequate	*P*-value	Non-random	100	Parents	**-0.332** (-0.532 – -0.132)	-
	LeCroy and Judy (2011)	Doubtful	*P*-value	Random	92	Mothers	**-3.323** (-3.761 – -2.885)	-
	Maher et al. (2011)	Very good	Effect size	Random	442	Parents	**0.003** (-0.09 – 0.096)	-
	Marcynyszyn et al. (2011)	Doubtful	*P*-value	Non-random	24	Caregivers	**0.059** (-0.328 – 0.446)	-
	McKelvey et al. (2012)	Inadequate	*P*-value	Non-random	93	Adolescent mothers	**-0.301** (-0.557 – -0.045)	-
	Miller et al. (2014)	Doubtful	*P*-value	Non-random	22	Mother	**-0.201** (-0.609 – 0.206)	-
	Renzaho and Sonia (2011)	Doubtful	*P*-value	Non-random	39	Parents	**0.424** (0.109 – 0.74)	-
	Robbers (2008)	Doubtful	*P*-value	Non-random	194	Adolescent parents	**0.92** (0.795 – 1.045)	+
	Rodriguez et al. (2010)	Adequate	*P*-value	Random	255	Mothers	**0.083** (-0.064 – 0.23)	-
	Schilling et al. (2017)	Adequate	*P*-value	Random	80	Parents	**0.206** (-0.013 – 0.425)	-
	Strickler et al. (2018)	Very good	Effect size	Non-random	66	Foster parents	**0.072** (-0.166 – 0.311)	-
	Twomey et al. (2010)	Doubtful	*P*-value	Non-random	52	Mothers	**-0.443** (-0.724 – -0.162)	-
	Waterston et al. (2009)	Very good	Effect size	Random	81	First-time mothers	**0.131** (-0.085 – 0.348)	-
	Wood et al. (2020)	Adequate	Effect size	Random	105	Caregivers	**0.528** (0.325 – 0.731)	+
	Zolnoski et al. (2012)	Adequate	*P*-value	Non-random	13	Parents	**0.18** (-0.333 – 0.694)	-
**AAPI-2: Role Reversal subscale**	Akai et al. (2008)	Adequate	*P*-value	Random	23	Mothers	**1.838** (0.67 – 3.005)	+
	Alvarez et al. (2018)	Very good	Effect size	Non-random	133	Parents	**0.25** (0.078 – 0.421)	-
	Barden et al. (2015)	Doubtful	*P*-value	Non-random	140	Couples with children	**0.189** (0.022 – 0.355)	-
	Benzies et al. (2011)	Doubtful	*P*-value	Non-random	23	Caregivers	**0.22** (-0.151 – 0.591)	-
	Benzies et al. (2014)	Very good	Effect size	Non-random	67	Parents	**0.249** (0.006 – 0.491)	-

(Continued)

Table S7. *(Continued).*

Measure: Overall scale / subscale[a]	Reference	Methodological quality of study[b]	Statistical method of study[c]	Sample allocation[d]	Sample size	Study population	Result of each study Hedges' *g* effect size[e] (95% CI)	Rating[f] on result per study
**AAPI-2: Role Reversal subscale**	Burton et al. (2018)	Very good	Effect size	Random	20	Parents	**-0.175** (-0.6 – 0.249)	-
	Clark et al. (2013)	Doubtful	Effect size	Non-random	69	Couples with babies	**1.263** (0.949 – 1.578)	+
	Conn et al. (2018)	Doubtful	*P*-value	Random	16	Foster parents	**-0.966** (-1.539 – -0.393)	-
	Conners et al. (2006)	Doubtful	*P*-value	Non-random	200	Mothers	**0.397** (0.254 – 0.541)	-
	Cullen et al. (2010)	Doubtful	*P*-value	Non-random	55	Mothers	**1.847** (1.415 – 2.28)	+
	Estefan et al. (2013)	Doubtful	*P*-value	Non-random	94	Parents	**0.849** (0.679 – 1.018)	+
	Galanter et al. (2012)	Adequate	Effect size	Non-random	48	Parents	**0.623** (0.318 – 0.928)	+
	Gibbs et al. (2008)	Inadequate	*P*-value	Non-random	100	Parents	**0.425** (0.222 – 0.628)	-
	LeCroy and Judy (2011)	Doubtful	*P*-value	Random	92	Mothers	**0.448** (0.161 – 0.735)	-
	Maher et al. (2011)	Very good	Effect size	Random	442	Parents	**-0.031** (-0.124 – 0.063)	-
	Marcynyszyn et al. (2011)	Doubtful	*P*-value	Non-random	24	Caregivers	**-0.071** (-0.459 – 0.316)	-
	McKelvey et al. (2012)	Inadequate	*P*-value	Non-random	93	Adolescent mothers	**0.172** (-0.083 – 0.427)	-
	Miller et al. (2014)	Doubtful	*P*-value	Non-random	22	Mother	**0.027** (-0.376 – 0.43)	-
	Renzaho and Sonia (2011)	Doubtful	*P*-value	Non-random	39	Parents	**0.759** (0.414 – 1.104)	+
	Robbers (2008)	Doubtful	*P*-value	Non-random	194	Adolescent parents	**0.169** (0.069 – 0.27)	-
	Rodriguez et al. (2010)	Adequate	*P*-value	Random	255	Mothers	**0.024** (-0.124 – 0.171)	-
	Schilling et al. (2017)	Adequate	*P*-value	Random	80	Parents	**0.583** (0.348 – 0.819)	+
	Strickler et al. (2018)	Very good	Effect size	Non-random	66	Foster parents	**0.082** (-0.157 – 0.321)	-
	Twomey et al. (2010)	Doubtful	*P*-value	Non-random	52	Mothers	**0.614** (0.321 – 0.906)	+
	Waterston et al. (2009)	Very good	Effect size	Random	81	First-time mothers	**0.367** (0.144 – 0.286)	-
	Wood et al. (2020)	Adequate	Effect size	Random	105	Caregivers	**0.096** (-0.094 – 0.591)	-
	Zolnoski et al. (2012)	Adequate	*P*-value	Non-random	13	Parents	**0.06** (-0.45 – 0.569)	-
	Burton et al. (2018)	Very good	Effect size	Random	20	Parents	**-0.175** (-0.6 – 0.249)	-
	Clark et al. (2013)	Doubtful	Effect size	Non-random	69	Couples with babies	**1.263** (0.949 – 1.578)	+
	Conn et al. (2018)	Doubtful	*P*-value	Random	16	Foster parents	**-0.966** (-1.539 – -0.393)	-
	Conners et al. (2006)	Doubtful	*P*-value	Non-random	200	Mothers	**0.397** (0.254 – 0.541)	-
	Cullen et al. (2010)	Doubtful	*P*-value	Non-random	55	Mothers	**1.847** (1.415 – 2.28)	+
	Estefan et al. (2013)	Doubtful	*P*-value	Non-random	94	Parents	**0.849** (0.679 – 1.018)	+
	Galanter et al. (2012)	Adequate	Effect size	Non-random	48	Parents	**0.623** (0.318 – 0.928)	+
	Gibbs et al. (2008)	Inadequate	*P*-value	Non-random	100	Parents	**0.425** (0.222 – 0.628)	-
	LeCroy and Judy (2011)	Doubtful	*P*-value	Random	92	Mothers	**0.448** (0.161 – 0.735)	-
	Maher et al. (2011)	Very good	Effect size	Random	442	Parents	**-0.031** (-0.124 – 0.063)	-

(Continued)

Table S7. *(Continued).*

Measure: Overall scale / subscale[a]	Reference	Methodological quality of study[b]	Statistical method of study[c]	Sample allocation[d]	Sample size	Study population	Result of each study Hedges' *g* effect size[e] (95% CI)	Rating[f] on result per study
**AAPI-2: Value of Corporal Punishment subscale**	Akai et al. (2008)	Adequate	*P*-value	Random	23	Mothers	**1.694** (0.569 – 2.818)	+
	Alvarez et al. (2018)	Very good	Effect size	Non-random	133	Parents	**0.125** (-0.044 – 0.295)	-
	Benzies et al. (2011)	Doubtful	*P*-value	Non-random	23	Caregivers	**-0.072** (-0.498 – 0.353)	-
	Benzies et al. (2014)	Very good	Effect size	Non-random	67	Parents	**0.233** (-0.007 – 0.473)	-
	Burton et al. (2018)	Very good	Effect size	Random	20	Parents	**0.411** (-0.029 – 0.851)	-
	Clark et al. (2013)	Doubtful	Effect size	Non-random	69	Couples with babies	**1.564** (1.214 – 1.914)	+
	Conn et al. (2018)	Doubtful	*P*-value	Random	16	Foster parents	**0.18** (-0.289 – 0.649)	-
	Conners et al. (2006)	Doubtful	*P*-value	Non-random	200	Mothers	**-0.014** (-0.152 – 0.124)	-
	Cullen et al. (2010)	Doubtful	*P*-value	Non-random	55	Mothers	**1.3** (0.944 – 1.656)	+
	Estefan et al. (2013)	Doubtful	*P*-value	Non-random	94	Parents	**1.043** (0.867 – 1.219)	+
	Galanter et al. (2012)	Adequate	Effect size	Non-random	48	Parents	**0.591** (0.288 – 0.893)	+
	Gibbs et al. (2008)	Inadequate	*P*-value	Non-random	100	Parents	**0.234** (0.036 – 0.431)	-
	LeCroy and Judy (2011)	Doubtful	*P*-value	Random	92	Mothers	**0.367** (0.082 – 0.653)	-
	Maher et al. (2011)	Very good	Effect size	Random	442	Parents	**0.001** (-0.092 – 0.094)	-
	Marcynyszyn et al. (2011)	Doubtful	*P*-value	Non-random	24	Caregivers	**0.261** (-0.132 – 0.655)	-
	McKelvey et al. (2012)	Inadequate	*P*-value	Non-random	93	Adolescent mothers	**-0.175** (-0.43 – 0.08)	-
	Miller et al. (2014)	Doubtful	*P*-value	Non-random	22	Mother	**0.588** (0.149 – 1.026)	+
	Renzaho and Sonia (2011)	Doubtful	*P*-value	Non-random	39	Parents	**0.846** (0.491 – 1.201)	+
	Robbers (2008)	Doubtful	*P*-value	Non-random	194	Adolescent parents	**1.758** (1.596 – 1.92)	+
	Rodriguez et al. (2010)	Adequate	*P*-value	Random	255	Mothers	**0.094** (-0.053 – 0.241)	-
	Schilling et al. (2017)	Adequate	*P*-value	Random	80	Parents	**0.212** (-0.007 – 0.432)	-
	Scudder et al. (2014)	Very good	Effect size	Random	39	Mothers	**0.365** (-0.07 – 0.8)	-
	Strickler et al. (2018)	Very good	Effect size	Non-random	66	Foster parents	**0.511** (0.257 – 0.765)	+
	Waterston et al. (2009)	Very good	Effect size	Random	81	First-time mothers	**0.306** (0.085 – 0.527)	-
	Wood et al. (2020)	Adequate	Effect size	Random	105	Caregivers	**0.128** (-0.063 – 0.319)	-
	Zolnoski et al. (2012)	Adequate	*P*-value	Non-random	13	Parents	**-0.352** (-0.878 – 0.175)	-
**APT: Overall scale**	Holland and Holden (2016)	Very good	Effect size	Random	21	Mothers of young children	**1.078** (0.448 – 1.708)	+
**CNQ: Overall scale**	No study included	NE	NE	NE	NE	NE	**NE**	**NE**
**CNS-MMS: Overall scale**	No study included	NE	NE	NE	NE	NE	**NE**	**NE**
**CTS-ES: Overall scale**	No study included	NE	NE	NE	NE	NE	**NE**	**NE**
**CTSPC: Overall scale**	Dubowitz et al. (2012)	Very good	Effect size	Random	583	Mothers	**0.12** (0 – 0.24)	-
	Feinberg et al. (2016)	Very good	*P*-value	Random	169	Couples expecting their first child	**0.688** (0.469 – 0.908)	+

(Continued)

Table S7. *(Continued).*

Measure: Overall scale / subscale[a]	Reference	Methodological quality of study[b]	Statistical method of study[c]	Sample allocation[d]	Sample size	Study population	Result of each study Hedges' *g* effect size[e] (95% CI)	Rating[f] on result per study
**CTSPC: Overall scale**	Fowler and Michael (2017)	Adequate	Effect size	Random	68	Parents	**NR**	?
	Guterman et al. (2013)	Adequate	Effect size	Random	73	Parents	**0.28** (-0.044 – 0.604)	-
	Guterman et al. (2018)	Adequate	Effect size	Non-random	23	Parents	**0.229** (-0.156 – 0.614)	-
	Knox and Burkhart (2014)	Very good	Effect size	Non-random	60	Parents and caregivers	**0.368** (0.11 – 0.627)	-
	Lindhiem et al. (2014)	Very good	Effect size	Non-random	139	Parents	**0.588** (0.348 – 0.828)	+
	McDonell et al. (2015)	Very good	*P*-value	Random	229	Parents or caregivers	**NR**	?
	Ondersma et al. (2017)	Adequate	Effect size	Random	112	Mothers	**0.026** (-0.149 – 0.201)	-
	Oveisi et al. (2010)	Adequate	Effect size	Random	108	Mothers	**0.407** (0.212 – 0.602)	-
	Portnoy et al. (2018)	Very good	*P*-value	Random	94	Caregivers	**0.138** (-0.144 – 0.42)	-
	Self-brown et al. (2017)	Adequate	Effect size	Random	50	Fathers	**0.777** (0.408 – 1.147)	+
	Shaffer et al. (2013)	Very good	Effect size	Random	137	Parents	**0.689** (0.446 – 0.931)	+
	Swenson et al. (2010)	Very good	*P*-value	Random	43	Parents	**0.469** (0.044 – 0.894)	-
	Wieling et al. (2015)	Doubtful	Effect size	Non-random	14	Mothers	**0.737** (0.173 – 1.301)	+
	Zoysa et al. (2015)	Adequate	*P*-value	Non-random	157	Parents	**0.372** (0.149 – 0.594)	-
**CTSPC: Physical Assault subscale**	Dubowitz et al. (2012)	Very good	Effect size	Random	583	Mothers	**0.154** (0.034 – 0.274)	-
	Feinberg et al. (2016)	Very good	Effect size	Random	169	Couples expecting their first child	**0.619** (0.401 – 0.836)	+
	Guterman et al. (2013)	Adequate	Effect size	Random	73	Parents	**0.302** (-0.022 – 0.627)	-
	Guterman et al. (2018)	Adequate	Effect size	Non-random	23	Parents	**0.276** (-0.111 – 0.663)	-
	Lindhiem et al. (2014)	Very good	Effect size	Non-random	139	Parents	**0.848** (0.603 – 1.093)	+
	Portnoy et al. (2018)	Very good	*P*-value	Random	94	Parents	**0.331** (0.048 – 0.614)	-
	Self-brown et al. (2017)	Adequate	Effect size	Random	50	Fathers	**0.31** (0.031 – 0.59)	-
	Shaffer et al. (2013)	Very good	Effect size	Random	137	Parents	**0.683** (0.441 – 0.925)	+
	Swenson et al. (2010)	Very good	Effect size	Random	43	Parents	**0.565** (0.138 – 0.992)	+
	Zoysa et al. (2015)	Adequate	*P*-value	Non-random	157	Parents	**0.349** (0.127 – 0.572)	-
**FM-CA: Overall scale**	Slep et al. (2020)	Adequate	*P*-value	Random	11377	Parents	**0.603** (0.582 – 0.624)	+
**ICAST-Trial: Overall scale**	Cluver et al. (2018)	Very good	*P*-value	Random	270	Caregivers	**0.392** (0.268 – 0.516)	-
	Lachman et al. (2020)	Very good	*P*-value	Random	248	Parents	**0.536** (0.442 – 0.63)	+
	Meinick et al. (2018)	Adequate	Effect size	Random	240	Primary caregivers	**0.31** (0.181 – 0.44)	-
	Shenderovich et al. (2019)	Doubtful	Effect size	Random	270	Caregivers	**0.303** (0.181 – 0.425)	-
**ICAST-Trial: Emotional Abuse subscale**	Lachman et al. (2020)	Very good	*P*-value	Random	248	Parents	**0.485** (0.392 – 0.578)	-
	Meinick et al. (2018)	Adequate	Effect size	Random	240	Primary caregivers	**0.32** (0.191 – 0.45)	-

(Continued)

Table S7. *(Continued).*

Measure: Overall scale / subscale[a]	Reference	Methodological quality of study[b]	Statistical method of study[c]	Sample allocation[d]	Sample size	Study population	Result of each study Hedges' *g* effect size[e] (95% CI)	Rating[f] on result per study
**ICAST-Trial: Neglect subscale**	Cluver et al. (2018)	Very good	Effect size	Random	270	Caregivers	**0.245** (0.124 – 0.366)	-
	Lachman et al. (2020)	Very good	Effect size	Random	248	Parents	**-0.02** (-0.108 – 0.069)	-
	Meinck et al. (2018)	Adequate	*P*-value	Random	240	Primary caregivers	**0.229** (0.101 – 0.357)	-
	Shenderovich et al. (2019)	Doubtful	*P*-value	Random	270	Caregivers	**0.21** (0.09 – 0.331)	-
**ICAST-Trial: Physical Abuse subscale**	Lachman et al. (2020)	Very good	Effect size	Random	248	Parents	**0.552** (0.458 – 0.647)	+
	Meinck et al. (2018)	Adequate	*P*-value	Random	240	Primary caregivers	**0.512** (0.378 – 0.647)	+
**ICAST-Trial: Sexual Abuse subscale**	Lachman et al. (2020)	Very good	Effect size	Random	248	Parents	**0.039** (-0.049 – 0.128)	-
	Meinck et al. (2018)	Adequate	*P*-value	Random	240	Primary caregivers	**0.179** (0.052 – 0.306)	-
**IPPS: Overall scale**	No study included	NE	NE	NE	NE	NE	**NE**	**NE**
**MCNS: Overall scale**	Gallitto et al. (2020)	Very good	Effect size	Non-random	68	Caregivers	**0.231** (-0.089 – 0.551)	-
**MCNS-SF: Overall scale**	No study included	NE	NE	NE	NE	NE	**NE**	**NE**
**P-CAAM: Overall scale**	No study included	NE	NE	NE	NE	NE	**NE**	**NE**
**POQ: Overall scale**	Sanders et al. (2004)	Very good	Effect size	Non-random	35	Parents	**0.866** (0.484 – 1.248)	+
	Vorhies et al. (2009)	Adequate	*P*-value	Non-random	17	Adolescent mothers	**0.86** (-0.088 – 1.492)	+
**PRCM: Overall scale**	Holland et al. (2016)	Very good	Effect size	Random	21	Mothers	**0.509** (-0.176 – 1.106)	-
	Caughy et al. (2003)	Doubtful	*P*-value	Random	134	Parents	**0.039** (-0.088 – 0.254)	+
**SBS-SV: Overall scale**	No study included	NE	NE	NE	NE	NE	**NE**	**NE**

Note. AAPI-2 = Adult Adolescent Parenting Inventory-2, APT = Analog Parenting Task, CNQ = Child Neglect Questionnaire, CNS-MMS = Child Neglect Scales-Maternal Monitoring and Supervision Scale, CTS-ES = Child Trauma Screen-Exposure Score, CTSPC = Conflict Tactics Scales: Parent-Child version, FM-CA = Family Maltreatment-Child Abuse criteria, ICAST-Trial = ISPCAN (International Society for the Prevention of Child Abuse and Neglect) Child Abuse Screening Tool for use in Trials, IPPS = Intensity of Parental Punishment Scale, MCNS = Mother-Child Neglect Scale, MCNS-SF = Mother-Child Neglect Scale-Short Form, P-CAAM = Parent-Child Aggression Acceptability Movie task, POQ = Parent Opinion Questionnaire, PRCM = Parental Response to Child Misbehavior questionnaire, SBS-SV = Shaken Baby Syndrome awareness assessment-Short Version; NE = Not Evaluated due to no intervention study assessing responsiveness, NR = Not Reported due to no relevant data found to calculate effect size.

[a] Subscales were included if data on factor analysis and Cronbach's alpha determined per subscale could be retrieved from the literature, thus confirming the scale's multidimensional structure (Mokkink et al., 2018).

[b] Methodological quality was evaluated using the Risk of Bias checklist for assessing the methodological quality of studies on responsiveness (Online Supplemental Table S3) in Step 2 of Figure 1.

[c] Statistical method for mean difference before and after intervention was used either to calculate *p*-values or to estimate effect sizes in the included studies. *P*-values were calculated through paired t-tests or repeated measures ANOVAs in most cases; effect size was estimated through calculating standardized mean differences (SMD) such as Cohen's *d* or Hedges' *g* (Hedges & Olkin, 2014).

[d] Random sample allocation indicates that the sample is randomly allocated to an intervention or control group; Non-random sample allocation indicates that the sample is not randomly allocated to an intervention or control group (Altman, 1991).

[e] Effect size was calculated using the formulas presented by Borenstein et al. (2009); Hedges' *g* = a statistic to measure the effect size from change scores between before and after intervention (Hedges & Olkin, 2014), CI = Confidence Interval.

[f] Rating on result of each study was determined using the criteria for good responsiveness (Online Supplemental Table S4) in Step 3.1 of Figure 1; + = Sufficient, ? = Indeterminate, - = Insufficient, ± = Inconsistent.

Table S8. *Pooled results, overall ratings, and quality of evidence on responsiveness per measure: Detailed findings for Step 3.2 and 3.3 in Figure 1.*

Measure	Overall scale / subscale[a]	Quality of evidence[b]				Pooled results	Overall Rating[e] on pooled results (overall rating on adjusted pooled results)	Overall quality of evidence[f] (reasons)
		Risk of bias	Inconsistency	Imprecision	Indirectness	Hedges' *g* effect size[c] (95% CI; I^2); Publication bias *p*-value[d] (adjusted effect size, 95% adjusted CI)		
**AAPI-2**	**Overall scale**	**No concern**: Multiple studies of adequate methodological quality	**Very serious concern**: High heterogeneity in results across studies (I^2 = 90%)	**No concern:** Pooled sample size = 4,430	**No concern:** All studies addressing target population of this review (caregiver or parent)	**0.397**(0.287 – 0.506; 90%); *p* = 0.061 (NA)	**- (NA)**	**Low** (totally inconsistent results across studies)
	**Inappropriate Expectations subscale**	**No concern**: Multiple studies of adequate methodological quality	**Very serious concern**: High heterogeneity in results across studies (I^2 = 92.4%)	**No concern:** Pooled sample size = 2,513	**No concern:** All studies addressing target population of this review (caregiver or parent)	**0.295** (0.143 – 0.447; 92.4%); *p* = 0.453 (NA)	**- (NA)**	**Low** (totally inconsistent results across studies)
	**Lack of Empathy subscale**	**No concern**: Multiple studies of adequate methodological quality	**Very serious concern**: High heterogeneity in results across studies (I^2 = 95.2%)	**No concern:** Pooled sample size = 2,758	**No concern:** All studies addressing target population of this review (caregiver or parent)	**0.392** (0.208 – 0.577; 95.2%); *p* = 0.021 (-0.012, -0.212 – 0.189)	**- (-)**	**Low** (totally inconsistent results across studies)
	**Oppressing Children's Power and Independence subscale**	**No concern**: Multiple studies of adequate methodological quality	**Very serious concern**: High heterogeneity in results across studies (I^2 = 96.2%)	**No concern:** Pooled sample size = 2,335	**No concern:** All studies addressing target population of this review (caregiver or parent)	**0.017** (-0.204 – 0.238; 96.2%); *p* = 0.747 (NA)	**- (NA)**	**Low** (totally inconsistent results across studies)
	**Role Reversal subscale**	**No concern**: Multiple studies of adequate methodological quality	**Very serious concern**: High heterogeneity in results across studies (I^2 = 90.1%)	**No concern:** Pooled sample size = 2,546	**No concern:** All studies addressing target population of this review (caregiver or parent)	**0.351** (0.216 – 0.486; 90.1%); *p* = 0.359 (NA)	**- (NA)**	**Low** (totally inconsistent results across studies)
	**Value of Corporal Punishment subscale**	**No concern**: Multiple studies of adequate methodological quality	**Very serious concern**: High heterogeneity in results across studies (I^2 = 95.7%)	**No concern:** Pooled sample size = 2,393	**No concern:** All studies addressing target population of this review (caregiver or parent)	**0.448** (0.231 – 0.665; 95.7%); *p* = 0.255 (NA)	**- (NA)**	**Low** (totally inconsistent results across studies)
**APT**	**Overall scale**	**No concern**: One study of very good methodological quality	**No concern**: Low heterogeneity in results across studies (I^2 = 0%)	**Very serious concern:** Pooled sample size = 21	**No concern:** All studies addressing target population of this review (caregiver or parent)	**1.078** (0.448 – 1.708; 0%); NA	**+ (NA)**	**Low** (very small total sample size)
**CNQ**	**Overall scale**	NE	NE	NE	NE	NE	NE	NE
**CNS-MMS**	**Overall scale**	NE	NE	NE	NE	NE	NE	NE
**CTS-ES**	**Overall scale**	NE	NE	NE	NE	NE	NE	NE

(Continued)

Table S8. *(Continued).*

Measure	Overall scale / subscale[a]	Quality of evidence[b]				Pooled results	Overall Rating[e] on pooled results (overall rating on adjusted pooled results)	Overall quality of evidence[f] (reasons)
		Risk of bias	Inconsistency	Imprecision	Indirectness	Hedges' *g* effect size[c] (95% CI; I^2); Publication bias *p*-value[d] (adjusted effect size, 95% adjusted CI)		
**CTSPC**	**Overall scale**	**No concern**: Multiple studies of adequate methodological quality	**Serious concern**: High heterogeneity in results across studies (I^2 = 77.4%)	**No concern:** Pooled sample size = 1,812	**No concern:** All studies addressing target population of this review (caregiver or parent)	**0.400** (0.260 – 0.539; 77.4%); *p* = 0.584 (NA)	**- (NA)**	**Low** (totally inconsistent results across studies)
	**Physical Assault subscale**	**No concern**: Multiple studies of adequate methodological quality	**Serious concern**: High heterogeneity in results across studies (I^2 = 77.4%)	**No concern:** Pooled sample size = 885	**No concern:** All studies addressing target population of this review (caregiver or parent)	**0.442** (0.277 – 0.607; 77.4%); *p* = 0.858 (NA)	**- (NA)**	**Low** (totally inconsistent results across studies)
**FM-CA**	**Overall scale**	**Serious concern**: Only one study of adequate methodological quality available	**No concern**: Low heterogeneity in results across studies (I^2 = 0%)	**No concern:** Pooled sample size = 11,377	**No concern:** All studies addressing target population of this review (caregiver or parent)	**0.603** (0.582 – 0.624; 0%); NA	**+ (NA)**	**Moderate** (only one study of adequate methodological quality available)
**ICAST-Trial**	**Overall scale**	**No concern**: Multiple studies of adequate methodological quality	**Very serious concern**: High heterogeneity in results across studies (I^2 = 75.5%)	**No concern:** Pooled sample size = 1,028	**No concern:** All studies addressing target population of this review (caregiver or parent)	**0.390** (0.273 – 0.508; 75.5%); *p* = 0.734 (NA)	**- (NA)**	**Low** (totally inconsistent results across studies)
	**Emotional Abuse subscale**	**No concern**: Multiple studies of adequate methodological quality	**Very serious concern**: High heterogeneity in results across studies (I^2 = 75.8%)	**No concern:** Pooled sample size = 488	**No concern:** All studies addressing target population of this review (caregiver or parent)	**0.409** (0.248 – 0.571; 75.8%); NA	**- (NA)**	**Low** (totally inconsistent results across studies)
	**Neglect subscale**	**No concern**: Multiple studies of adequate methodological quality	**Very serious concern**: High heterogeneity in results across studies (I^2 = 83.9%)	**No concern:** Pooled sample size = 1,028	**No concern:** All studies addressing target population of this review (caregiver or parent)	**0.162** (0.021 – 0.302; 83.9%); *p* = 0.308 (NA)	**- (NA)**	**Low** (totally inconsistent results across studies)
	**Physical Abuse subscale**	**No concern**: Multiple studies of adequate methodological quality	**No concern**: High heterogeneity in results across studies (I^2 = 0%)	**No concern:** Pooled sample size = 488	**No concern:** All studies addressing target population of this review (caregiver or parent)	**0.539** (0.462 – 0.616; 0%); NA	**+ (NA)**	**High** (no concern)
	**Sexual Abuse subscale**	**No concern**: Multiple studies of adequate methodological quality	**Serious concern**: Moderate heterogeneity in results across studies (I^2 = 68.0%)	**No concern:** Pooled sample size = 488	**No concern:** All studies addressing target population of this review (caregiver or parent)	**0.102** (-0.034 – 0.238; 68.0%); NA	**- (NA)**	**Moderate** (partly inconsistent results across studies)
**IPPS**	**Overall scale**	NE	NE	NE	NE	NE	NE	NE

(Continued)

Table S8. *(Continued).*

Measure	Overall scale / subscale[a]	Quality of evidence[b]				Pooled results	Overall Rating[e] on pooled results (overall rating on adjusted pooled results)	Overall quality of evidence[f] (reasons)
		Risk of bias	Inconsistency	Imprecision	Indirectness	Hedges' *g* effect size[c] (95% CI; I^2); Publication bias *p*-value[d] (adjusted effect size, 95% adjusted CI)		
**MCNS**	**Overall scale**	**No concern**: One study of very good methodological quality	**No concern**: Low heterogeneity in results across studies (I^2 = 0%)	**Serious concern:** Pooled sample size = 68	**No concern:** All studies addressing target population of this review (caregiver or parent)	**0.231** (-0.089 – 0.551; 0%); NA	**- (NA)**	**Moderate** (small total sample size)
**MCNS-SF**	**Overall scale**	NE	NE	NE	NE	NE	NE	NE
**P-CAAM**	**Overall scale**	NE	NE	NE	NE	NE	NE	NE
**POQ**	**Overall scale**	**No concern**: One study of very good methodological quality	**No concern**: Low heterogeneity in results across studies (I^2 = 0%)	**Serious concern:** Pooled sample size = 52	**No concern:** All studies addressing target population of this review (caregiver or parent)	**0.864** (0.537 – 1.191; 0%); NA	**+ (NA)**	**Moderate** (small total sample size)
**PRCM**	**Overall scale**	**No concern**: One study of very good methodological quality	**Serious concern**: Moderate heterogeneity in results across studies (I^2 = 52.7%)	**No concern:** Pooled sample size = 155	**No concern:** All studies addressing target population of this review (caregiver or parent)	**0.188** (-0.241 – 0.618; 52.7%); NA	**- (NA)**	**Moderate** (partly inconsistent results across studies)
**SBS-SV**	**Overall scale**	NE	NE	NE	NE	NE	NE	NE

Note. AAPI-2 = Adult Adolescent Parenting Inventory-2, APT = Analog Parenting Task, CNQ = Child Neglect Questionnaire, CNS-MMS = Child Neglect Scales-Maternal Monitoring and Supervision Scale, CTS-ES = Child Trauma Screen-Exposure Score, CTSPC = Conflict Tactics Scales: Parent-Child version, FM-CA = Family Maltreatment-Child Abuse criteria, ICAST-Trial = ISPCAN (International Society for the Prevention of Child Abuse and Neglect) Child Abuse Screening Tool for use in Trials, IPPS = Intensity of Parental Punishment Scale, MCNS = Mother-Child Neglect Scale, MCNS-SF = Mother-Child Neglect Scale-Short Form, P-CAAM = Parent-Child Aggression Acceptability Movie task, POQ = Parent Opinion Questionnaire, PRCM = Parental Response to Child Misbehavior questionnaire, SBS-SV = Shaken Baby Syndrome awareness assessment-Short Version; NE = Not Evaluated due to no intervention study assessing responsiveness; NA = Not Applicable due to there being either a limited number of studies or non-significant publication bias.

[a] Subscales were included if data on factor analysis and Cronbach's alpha determined per subscale could be retrieved from the literature, thus confirming the scale's multidimensional structure (Mokkink et al., 2018).

[b] Quality of evidence consists of four factors: risk of bias (methodological quality of the studies: step 2 in Figure 1), inconsistency (inconsistent results across the studies: final pooled results from step 3.2 in Figure 1), Imprecision (small pooled sample size of the studies resulting in wide confidence intervals), and indirectness (evidence from different populations other than the ones of interest in the review).

[c] Effect size was calculated using the formulas presented by Borenstein et al. (2009); Hedges' *g* = a statistic to measure the effect size from change scores between before and after intervention (Hedges & Olkin, 2014), CI = Confidence Interval, I^2 = *I-squared* as measure of inconsistency (the percentage of total variability across studies due to heterogeneity; Higgins et al., 2003).

[d] Publication bias refers to the bias that may inflate the pooled effects as studies with small sample sizes and small effects may potentially be unpublished and missing (Higgins & Green, 2011); A publication bias *p*-value obtained by Begg's test (Begg & Mazumdar, 1994) of less than 0.05 indicated significant publication bias existed in the pooled effect size; If publication bias was indicated, then the trim-and-fill test by Duval and Tweedie (2000) was next performed using the fixed-effect model to produce an adjusted pooled effect size and confidence interval after accounting for missing studies due to publication bias (Duval & Tweedie, 2000); The publication bias were not tested with less than three studies (Higgins & Green, 2011).

[e] Overall rating on pooled result of all studies was determined using the criteria for good responsiveness (Online Supplemental Table S4) in the step 3.2 of Figure 1; + = Sufficient, ? = Indeterminate, - = Insufficient, ± = Inconsistent; The same criteria were applied to determine overall rating on adjusted pooled results accounting for missing studies due to publication bias (Duval & Tweedie, 2000).

[f] Overall quality of evidence was downgraded using the modified GRADE approach (Online Supplemental Table S5) for grading the quality of summarized evidence on responsiveness (Step 3.3 of Figure 1) when there were concerns regarding each factor on quality of evidence: High = high level of confidence in overall ratings, Moderate = moderate level of confidence in overall ratings, Low = low level of confidence in overall ratings, Very Low = very low level of confidence in overall ratings; Publication bias was not considered for grading the quality of evidence in the modified GRADE approach due to a lack of registries for studies on psychometric properties according to the COSMIN manual (Mokkink, Prinsen, et al., 2018).

References for Online Supplemental Materials

Akai, C. E., Guttentag, C. L., Baggett, K. M., & Noria, C. C. (2008). Enhancing parenting practices of at-risk mothers. *The Journal of Primary Prevention, 29*(3), 223-242. https://doi.org/10.1007/s10935-008-0134-z

Altman, D. G. (1991). *Practical statistics for medical research*. Chapman and Hall.

Alvarez, M., Rodrigo, M. J., & Byrne, S. (2018). What implementation components predict positive outcomes in a parenting program?. *Research on Social Work Practice, 28*(2), 173-187. https://doi.org/10.1177/1049731516640903

Axford, N., Bjornstad, G., Matthews, J., Heilmann, S., Raja, A., Ukoumunne, O. C., Berry, V., Wilkinson, T., Timmons, L., Hobbs, T., Eames, T., Kallitsoglou, A., Blower, S., & Warner, G. (2020). The effectiveness of a therapeutic parenting program for children aged 6-11 years with behavioral or emotional difficulties: Results from a randomized controlled trial. *Children and Youth Services Review, 117*. https://doi.org/10.1016/j.childyouth.2020.105245

Barden, S. M., Carlson, R. G., Daire, A. P., Finnell, L. R., Christopher, K., & Young, E. (2015). Investigating the influence of relationship education on parental attitudes. *Marriage & Family Review*, *51*(3), 246-263. https://doi.org/10.1080/01494929.2015.1031422

Barnes, J., Stuart, J., Allen, E., Petrou, S., Sturgess, J., Barlow, J., Macdonald, G., Spiby, H., Aistrop, D., Melhuish, E., Kim, S. W., & Elbourne, D. (2017). Randomized controlled trial and economic evaluation of nurse-led group support for young mothers during pregnancy and the first year postpartum versus usual care. *Trials, 18*(1), 508. https://doi.org/10.1186/s13063-017-2259-y

Barnet, B., Liu, J., DeVoe, M., Alperovitz-Bichell, K., & Duggan, A. K. (2007). Home visiting for adolescent mothers: Effects on parenting, maternal life course, and

primary care linkage. *Annals of Family Medicine*, *5*(3), 224-232. https://doi.org/10.1370/afm.629

Begg, C. B., & Mazumdar, M. (1994). Operating Characteristics of a Rank Correlation Test for Publication Bias. *Biometrics, 50*(4), 1088-1101. https://doi.org/10.2307/2533446

Benzies, K., Mychasiuk, R., Kurilova, J., Tough, S., Edwards, N., & Donnelly, C. (2014). Two-generation preschool programme: Immediate and 7-year-old outcomes for low-income children and their parents. *Child & Family Social Work*, *19*(2), 203-214. https://doi.org/10.1111/j.1365-2206.2012.00894.x

Benzies, K., Tough, S., Edwards, N., Mychasiuk, R., & Donnelly, C. (2011). Aboriginal Children and Their Caregivers Living with Low Income: Outcomes from a Two-Generation Preschool Program. *Journal of Child and Family Studies*, *20*(3), 311-318. https://doi.org/10.1007/s10826-010-9394-3

Berry, M., McCauley, K., & Lansing, T. (2007). Permanency through group work: A pilot intensive reunification program. *Child & Adolescent Social Work Journal*, *24*(5), 477-493. https://doi.org/10.1007/s10560-007-0102-0

Borenstein, M., Hedges, L. V., Higgins, J., & Rothstein, H. R. (2009). Introductionto meta-analysis. *West Sussex, England: John Wiley&Sons Ltd*.

Burton, R. S., Zwahr-Castro, J., Magrane, C. L., Hernandez, H., Farley, L. G., & Amodei, N. (2018). The Nurturing Program: An Intervention for Parents of Children with Special Needs. *Journal of Child and Family Studies*, *27*(4), 1137-1149. https://doi.org/10.1007/s10826-017-0966-3

Caughy, M. O. B., Miller, T. L., Genevro, J. L., Huang, K.-Y., & Nautiyal, C. (2003). The effects of Healthy Steps on discipline strategies of parents of young children. *Journal of Applied Developmental Psychology*, *24*(5), 517-534. https://doi.org/10.1016/j.appdev.2003.08.004

Clark, C., Young, M., & Dow, M. G. (2013). Can strengthening parenting couples' relationships reduce at-risk parenting attitudes?. *The Family Journal*, *21*(3), 306-312. https://doi.org/10.1177/1066480713476841

Cluver, L. D., Meinck, F., Steinert, J. I., Shenderovich, Y., Doubt, J., Herrero Romero, R., Lombard, C. J., Redfern, A., Ward, C. L., Tsoanyane, S., Nzima, D., Sibanda, N., Wittesaele, C., De Stone, S., Boyes, M. E., Catanho, R., Lachman, J. M., Salah, N., Nocuza, M., & Gardner, F. (2018). Parenting for Lifelong Health: a pragmatic cluster randomised controlled trial of a non-commercialised parenting programme for adolescents and their families in South Africa. *BMJ Global Health*, *3*(1), e000539. https://doi.org/10.1136/bmjgh-2017-000539

Cohen, J. (1988). *Statistical power analysis for the behavioral sciences*. Academic Press.

Conn, A.-M., Szilagyi, M. A., Alpert-Gillis, L., Webster-Stratton, C., Manly, J. T., Goldstein, N., & Jee, S. H. (2018). Pilot randomized controlled trial of foster parent training: A mixed-methods evaluation of parent and child outcomes. *Children and Youth Services Review*, *89*, 188-197. https://doi.org/10.1016/j.childyouth.2018.04.035

Conners, N. A., Grant, A., Crone, C. C., & Whiteside-Mansell, L. (2006). Substance abuse treatment for mothers: Treatment outcomes and the impact of length of stay. *Journal of Substance Abuse Treatment, 31*(4), 447-456. https://doi.org/10.1016/j.jsat.2006.06.001

Cordier, R., Speyer, R., Chen, Y. W., Wilkes-Gillan, S., Brown, T., Bourke-Taylor, H., Doma, K., & Leicht, A. (2015). Evaluating the Psychometric Quality of Social Skills Measures: A Systematic Review. *PloS One, 10*(7), e0132299. https://doi.org/10.1371/journal.pone.0132299

Cullen, J. P., Ownbey, J. B., & Ownbey, M. A. (2010). The effects of the Healthy Families America home visitation program on parenting attitudes and practices and child social and emotional competence. *Child & Adolescent Social Work Journal*, *27*(5), 335-354. https://doi.org/10.1007/s10560-010-0206-9

Dubowitz, H., Lane, W. G., Semiatin, J. N., & Magder, L. S. (2012). The SEEK model of pediatric primary care: can child maltreatment be prevented in a low-risk population? *Academic Pediatrics, 12*(4), 259-268. https://doi.org/10.1016/j.acap.2012.03.005

Duval, S., & Tweedie, R. (2000). Trim and Fill: A Simple Funnel-Plot–Based Method of Testing and Adjusting for Publication Bias in Meta-Analysis. *Biometrics, 56*(2), 455-463. https://doi.org/10.1111/j.0006-341X.2000.00455.x

Estefan, L. F., Coulter, M. L., VandeWeerd, C. L., Armstrong, M., & Gorski, P. (2013). Relationships between stressors and parenting attitudes in a child welfare parenting program. *Journal of Child and Family Studies*, *22*(2), 199-208. https://doi.org/10.1007/s10826-012-9569-1

Farber, M. L. (2009). Parent mentoring and child anticipatory guidance with Latino and African American families. *Health & Social Work, 34*(3), 179-189. https://doi.org/10.1093/hsw/34.3.179

Feinberg, M. E., Jones, D. E., Hostetler, M. L., Roettger, M. E., Paul, I. M., & Ehrenthal, D. B. (2016). Couple-focused prevention at the transition to parenthood, a randomized trial: Effects on coparenting, parenting, family violence, and parent and child adjustment. *Prevention Science, 17*(6), 751-764. https://doi.org/10.1007/s11121-016-0674-z

Fowler, P. J., & Schoeny, M. (2017). Permanent housing for child welfare-involved families: Impact on child maltreatment overview. *American Journal of Community Psychology*, *60*(1-2), 91-102. https://doi.org/10.1002/ajcp.12146

Galanter, R., Self-Brown, S., Valente, J. R., Dorsey, S., Whitaker, D. J., Bertuglia-Haley, M., & Prieto, M. (2012). Effectiveness of parent-child interaction therapy delivered to at-risk families in the home setting. *Child & Family Behavior Therapy*, *34*(3), 177-196. https://doi.org/10.1080/07317107.2012.707079

Gallitto, E., Romano, E., & Whitaker, D. (2020). Investigating the Impact of the SafeCare Program on Parenting Behaviours in Child Welfare-Involved Families. *Child and Adolescent Social Work Journal*. https://doi.org/10.1007/s10560-020-00672-6

Gibbs, A., Moor, S., Frampton, C., & Watkins, W. (2008). Impact of psychosocial interventions on children with disruptive and emotional disorders treated in a health camp. *Australian & New Zealand Journal of Psychiatry*, *42*(9), 789-799. https://doi.org/10.1080/00048670802277248

Guterman, N. B., Bellamy, J. L., & Banman, A. (2018). Promoting father involvement in early home visiting services for vulnerable families: Findings from a pilot study of "Dads Matter". *Child Abuse & Neglect*, *76*, 261-272. https://doi.org/10.1016/j.chiabu.2017.10.017

Guterman, N. B., Tabone, J. K., Bryan, G. M., Taylor, C. A., Napoleon-Hanger, C., & Banman, A. (2013). Examining the effectiveness of home-based parent aide services to reduce risk for physical child abuse and neglect: Six-month findings from a randomized clinical trial. *Child Abuse & Neglect*, *37*(8), 566-577. https://doi.org/10.1016/j.chiabu.2013.03.006

Hedges, L. V., & Olkin, I. (2014). *Statistical methods for meta-analysis*. Academic press.

Higgins, J. P. T., & Green, S. (Eds.). (2011). *Cochrane handbook for systematic reviews of interventions version 5.1.0 [updated March 2011]*. The Cochrane Collaboration. https://handbook-5-1.cochrane.org/front_page.htm.

Higgins, J. P. T., Thompson, S. G., Deeks, J. J., & Altman, D. G. (2003). Measuring inconsistency in meta-analyses. *BMJ*, *327*, 557. https://doi.org/10.1136/bmj.327.7414.557

Holland, G. W., & Holden, G. W. (2016). Changing orientations to corporal punishment: A randomized, control trial of the efficacy of a motivational approach to psycho-education. *Psychology of Violence*, *6*(2), 233-242. https://doi.org/10.1037/a0039606

Knox, M., & Burkhart, K. (2014). A multi-site study of the ACT Raising Safe Kids program: Predictors of outcomes and attrition. *Children and Youth Services Review*, *39*, 20-24. https://doi.org/10.1016/j.childyouth.2014.01.006

Lachman, J., Wamoyi, J., Spreckelsen, T., Wight, D., Maganga, J., & Gardner, F. (2020). Combining parenting and economic strengthening programmes to reduce violence against children: a cluster randomised controlled trial with predominantly male caregivers in rural Tanzania. *BMJ Glob Health*, *5*(7). https://doi.org/10.1136/bmjgh-2020-002349

Lavi, I., Gard, A. M., Hagan, M., Van Horn, P., & Lieberman, A. F. (2015). Child-Parent Psychotherapy examined in a perinatal sample: Depression, posttraumatic stress symptoms and child-rearing attitudes. *Journal of Social and Clinical Psychology*, *34*(1), 64-82. https://doi.org/10.1521/jscp.2015.34.1.64

Lawson, M. A., Alameda-Lawson, T., & Byrnes, E. C. (2012). A multilevel evaluation of a comprehensive child abuse prevention program. *Research on Social Work Practice*, *22*(5), 553-566. https://doi.org/10.1177/1049731512444165

LeCroy, C. W., & Krysik, J. (2011). Randomized trial of the healthy families Arizona home visiting program. *Children and Youth Services Review*, *33*(10), 1761-1766. https://doi.org/10.1016/j.childyouth.2011.04.036

Lindhiem, O., Shaffer, A., & Kolko, D. J. (2014). Quantifying discipline practices using absolute versus relative frequencies: Clinical and research implications for child welfare. *Journal of Interpersonal Violence*, *29*(1), 66-81. https://doi.org/10.1177/0886260513504650

Maher, E. J., Marcynyszyn, L. A., Corwin, T. W., & Hodnett, R. (2011). Dosage matters: The relationship between participation in the Nurturing Parenting Program for infants, toddlers, and preschoolers and subsequent child maltreatment. *Children and Youth Services Review*, *33*(8), 1426-1434. https://doi.org/10.1016/j.childyouth.2011.04.014

Marcynyszyn, L. A., Maher, E. J., & Corwin, T. W. (2011). Getting with the (evidence-based) program: An evaluation of the Incredible Years Parenting Training Program in child welfare. *Children and Youth Services Review, 33*(5), 747-757. https://doi.org/10.1016/j.childyouth.2010.11.021

McDonell, J. R., Ben-Arieh, A., & Melton, G. B. (2015). Strong Communities for Children: Results of a multi-year community-based initiative to protect children from harm. *Child Abuse & Neglect*, *41*, 79-96. https://doi.org/10.1016/j.chiabu.2014.11.016

McKelvey, L. M., Burrow, N. A., Balamurugan, A., Whiteside-Mansell, L., & Plummer, P. (2012). Effects of home visiting on adolescent mothers' parenting attitudes. *American Journal of Public Health, 102*(10), 1860-1862. https://doi.org/10.2105/AJPH.2012.300934

Meinck, F., Boyes, M. E., Cluver, L., Ward, C. L., Schmidt, P., DeStone, S., & Dunne, M. P. (2018). Adaptation and psychometric properties of the ISPCAN Child Abuse Screening Tool for use in trials (ICAST-Trial) among South African adolescents and their primary caregivers. *Child Abuse & Neglect*, *82*, 45-58. https://doi.org/10.1016/j.chiabu.2018.05.022

Miller, A. L., Weston, L. E., Perryman, J., Horwitz, T., Franzen, S., & Cochran, S. (2014). Parenting while incarcerated: Tailoring the Strengthening Families Program for use with jailed mothers. *Children and Youth Services Review*, *44*, 163-170. https://doi.org/10.1016/j.childyouth.2014.06.013

Mokkink, L. B., Prinsen, C. A. C., Patrick, D. L., Alonso, J., Bouter, L. M., de Vet, H. C. W., & Terwee, C. B. (2018). *COSMIN methodology for systematic reviews of Patient‐ Reported Outcome Measures (PROMs)-User manual (version 1.0).* https://www.cosmin.nl/wp-content/uploads/COSMIN-syst-review-for-PROMs-manual_version-1_feb-2018.pdf

Ondersma, S. J., Martin, J., Fortson, B., Whitaker, D. J., Self-Brown, S., Beatty, J., Loree, A., Bard, D., & Chaffin, M. (2017). Technology to augment early home visitation for child maltreatment prevention: A pragmatic randomized trial. *Child Maltreatment*, *22*(4), 334-343. https://doi.org/10.1177/1077559517729890

Oveisi, S., Ardabili, H. E., Dadds, M. R., Majdzadeh, R., Mohammadkhani, P., Rad, J. A., & Shahrivar, Z. (2010). Primary prevention of parent-child conflict and abuse in Iranian mothers: a randomized-controlled trial. *Child Abuse Negl*, *34*(3), 206-213. https://doi.org/10.1016/j.chiabu.2009.05.008

Palusci, V. J., Crum, P., Bliss, R., & Bavolek, S. J. (2008). Changes in parenting attitudes and knowledge among inmates and other at-risk populations after a family nurturing program. *Children and Youth Services Review*, *30*(1), 79-89. https://doi.org/10.1016/j.childyouth.2007.06.006

Portnoy, J., Raine, A., Liu, J., & Hibbeln, J. R. (2018). Reductions of intimate partner violence resulting from supplementing children with omega-3 fatty acids: A randomized, double-blind, placebo-controlled, stratified, parallel-group trial. *Aggressive Behavior*, *44*(5), 491-500. https://doi.org/10.1002/ab.21769

Renzaho, A. M. N., & Vignjevic, S. (2011). The impact of a parenting intervention in Australia among migrants and refugees from Liberia, Sierra Leone, Congo, and Burundi: Results from the African Migrant Parenting Program. *Journal of Family Studies*, *17*(1), 71-79. https://doi.org/10.5172/jfs.2011.17.1.71

Robbers, M. L. (2008). The caring equation: An intervention program for teenage mothers and their male partners. *Children & Schools*, *30*(1), 37-47. https://doi.org/10.1093/cs/30.1.37

Rodriguez, M. L., Dumont, K., Mitchell-Herzfeld, S. D., Walden, N. J., & Greene, R. (2010). Effects of Healthy Families New York on the promotion of maternal parenting competencies and the prevention of harsh parenting. *Child Abuse & Neglect*, *34*(10), 711-723. https://doi.org/10.1016/j.chiabu.2010.03.004

Sanders, M. R., Pidgeon, A. M., Gravestock, F., Connors, M. D., Brown, S., & Young, R. W. (2004). Does parental attributional retraining and anger management enhance the effects of the triple P-positive parenting program with parents at risk of child maltreatment? *Behavior Therapy*, *35*(3), 513-535. https://doi.org/10.1016/S0005-7894(04)80030-3

Sangalang, B. B., & Rounds, K. (2005). Differences in health behaviors and parenting knowledge between pregnant adolescents and parenting adolescents. *Social Work in Health Care*, *42*(2), 1-22. https://doi.org/10.1300/J010v42n02_01

Sawasdipanich, N., Srisuphan, W., Yenbut, J., Tiansawad, S., & Humphreys, J. C. (2010). Effects of a cognitive adjustment program for Thai parents. *Nursing & Health Sciences*, *12*(3), 306-313. https://doi.org/10.1111/j.1442-2018.2010.00531.x

Schilling, S., French, B., Berkowitz, S. J., Dougherty, S. L., Scribano, P. V., & Wood, J. N. (2017). Child-Adult Relationship Enhancement in Primary Care (PriCARE): A

Randomized Trial of a Parent Training for Child Behavior Problems. *Academic Pediatrics, 17*(1), 53-60. https://doi.org/10.1016/j.acap.2016.06.009

Scudder, A. T., McNeil, C. B., Chengappa, K., & Costello, A. H. (2014). Evaluation of an existing parenting class within a women's state correctional facility and a parenting class modeled from Parent-Child Interaction Therapy. *Children and Youth Services Review, 46*, 238-247. https://doi.org/10.1016/j.childyouth.2014.08.015

Self-brown, S., Osborne, M. C., Lai, B. S., De Veauuse Brown, N., Glasheen, T. L., & Adams, M. C. (2017). Initial Findings from a Feasibility Trial Examining the SafeCare Dad to Kids Program with Marginalized Fathers. *Journal of Family Violence*, *32*(8), 751-766. https://doi.org/10.1007/s10896-017-9940-5

Shaffer, A., Lindhiem, O., & Kolko, D. J. (2013). Treatment effects of a modular intervention for early-onset child behavior problems on family contextual outcomes. *Journal of Emotional and Behavioral Disorders*, *21*(4), 277-288. https://doi.org/10.1177/1063426612462742

Shenderovich, Y., Eisner, M., Cluver, L., Doubt, J., Berezin, M., Majokweni, S., & Murray, A. L. (2019). Delivering a parenting program in South Africa: The impact of implementation on outcomes. *Journal of Child and Family Studies*, *28*(4), 1005-1017. https://doi.org/10.1007/s10826-018-01319-y

Slep, A. M. S., Heyman, R. E., Lorber, M. F., Baucom, K. J. W., & Linkh, D. J. (2020). Evaluating the Effectiveness of NORTH STAR: a Community-Based Framework to Reduce Adult Substance Misuse, Intimate Partner Violence, Child Abuse, Suicidality, and Cumulative Risk. *Prevention Science, 21*(7), 949-959. https://doi.org/10.1007/s11121-020-01156-w

Stover, C. S., McMahon, T. J., & Moore, K. (2019). A randomized pilot trial of two parenting interventions for fathers in residential substance use disorder treatment.

Journal of Substance Abuse Treatment, *104*, 116-127. https://doi.org/10.1016/j.jsat.2019.07.003

Strickler, A., Trunzo, A. C., & Kaelin, M. S. (2018). Treatment foster care pre-service trainings: Changes in parenting attitudes and fostering readiness. *Child & Youth Care Forum*, *47*(1), 61-79. https://doi.org/10.1007/s10566-017-9418-x

Suess, G., Bohlen, U., Carlson, E., Spangler, G., & Frumentia Maier, M. (2016). Effectiveness of attachment based STEEPTM intervention in a German high-risk sample. *Attachment & Human Development*, *18*(5), 443-460. https://doi.org/10.1080/14616734.2016.1165265

Swenson, C. C., Schaeffer, C. M., Henggeler, S. W., Faldowski, R., & Mayhew, A. M. (2010). Multisystemic therapy for child abuse and neglect: A randomized effectiveness trial. *Journal of Family Psychology*, *24*(4), 497-507. https://doi.org/10.1037/a0020324

Thomas, D. V., & Looney, S. W. (2004). Effectiveness of a Comprehensive Psychoeducational Intervention With Pregnant and Parenting Adolescents: A Pilot Study. *Journal of Child and Adolescent Psychiatric Nursing*, *17*(2), 66-77. https://doi.org/10.1111/j.1744-6171.2004.00066.x

Twomey, J. E., Miller-Loncar, C., Hinckley, M., & Lester, B. M. (2010). After family treatment drug court: Maternal, infant, and permanency outcomes. *Child Welfare: Journal of Policy, Practice, and Program*, *89*(6), 23-41. https://pubmed.ncbi.nlm.nih.gov/21877562/

Vorhies, V., Glover, C. M., Davis, K., Hardin, T., Krzyzanowski, A., Harris, M., Fagan, M., & Wilkniss, S. (2009). Improving outcomes for pregnant and parenting foster care youth with severe mental illness: An evaluation of a transitional living program.

Psychiatric Rehabilitation Journal, 33(2), 115-124. https://doi.org/10.2975/33.2.2009.115.124

Waters, S. F., Hagan, M. J., Rivera, L., & Lieberman, A. F. (2015). Improvements in the child-rearing attitudes of Latina mothers exposed to interpersonal trauma predict greater maternal sensitivity toward their 6-month-old infants. *Journal of Traumatic Stress, 28*(5), 426-433. https://doi.org/10.1002/jts.22043

Waterston, T., Welsh, B., Keane, B., Cook, M., Hammal, D., Parker, L., & McConachie, H. (2009). Improving early relationships: A randomized, controlled trial of an age-paced parenting newsletter. *Pediatrics*, *123*(1), 241-247. https://doi.org/10.1542/peds.2007-1872

Wieling, E., Mehus, C., Mollerherm, J., Neuner, F., Achan, L., & Catani, C. (2015). Assessing the feasibility of providing a parenting intervention for war-affected families in Northern Uganda. *Family & Community Health: The Journal of Health Promotion & Maintenance, 38*(3), 252-267. https://doi.org/10.1097/FCH.0000000000000064

Wood, J. N., Kratchman, D., Scribano, P. V., Berkowitz, S., & Schilling, S. (2020). Improving Child Behaviors and Parental Stress: A Randomized Trial of Child Adult Relationship Enhancement in Primary Care. *Academic Pediatrics*. https://doi.org/10.1016/j.acap.2020.08.002

Zajicek-Farber, M. L. (2010). Building practice evidence for parent mentoring home visiting in early childhood. *Research on Social Work Practice, 20*(1), 46-64. https://doi.org/10.1177/1049731509333172

Zolnoski, S., Stacks, A. M., Kohl-Hanlon, A., & Dykehouse, T. A. (2012). Lessons learned from the first-year evaluation of a small-scale home visitation program. *Journal of*

Social Service Research, 38(4), 515-528. https://doi.org/10.1080/01488376.2012.699407

Zoysa, P., Siriwardhana, C., Samaranayake, M., Athukorala, S., Kumari, S., & Fernando, D. (2015). The Impact of an Awareness Raising Program to Reduce Parental use of Aversive Disciplinary Practices. *Journal of Family Violence, 30*(5), 651-659. https://doi.org/10.1007/s10896-015-9701-2
